# Trends on Strategies for Mitigation of Antibiotic Residues in Milk and Dairy Products Based on Scientometric Analysis and Systematic Review

**DOI:** 10.1111/1541-4337.70398

**Published:** 2026-01-16

**Authors:** Emelda Orlando Simbine‐Ribisse, Wilma Custódio Fumo, Eugénio da Piedade Edmundo Sitoe, Patrícia Aparecida de Campos Braga, Cristiano João Macuamule, Adriana Pavesi Arisseto Bragotto

**Affiliations:** ^1^ Department of Food Science and Nutrition, Faculty of Food Engineering Universidade Estadual de Campinas (UNICAMP) Campinas Brazil; ^2^ Department of Animal Production and Food Technology, Veterinary Faculty Universidade Eduardo Mondlane Maputo Mozambique; ^3^ Section of Pharmacology and Toxicology, Department of Public and Animal Health, Veterinary Faculty Universidade Eduardo Mondlane Maputo Mozambique; ^4^ Centre for Excellence in Agro‐Food Systems and Nutrition Universidade Eduardo Mondlane Maputo Mozambique

**Keywords:** antibiotic reduction, bibliometric, dairy products, nonthermal, thermal

## Abstract

The presence of antibiotic residues (ARs) in milk poses a significant challenge to public health and the dairy industry. This study presents the research trends on strategies to mitigate ARs in milk and dairy products, combining scientometric and systematic review approaches. Following the PRISMA guidelines, data from Web of Science, ScienceDirect, PubMed, Embase, AGRICOLA, and CAS Abstract yielded 49 original articles. Geographical analysis revealed a heterogeneous distribution, with Spain as the leading contributor. The analysis showed that heat treatment is the most studied mitigation strategy, reflecting its widespread industrial implementation. Crucially, a clear trend indicates the growth of emerging nonthermal technologies, such as pulsed electric field (PEF) and ozonation, driven by the need to preserve the nutritional and sensory attributes of milk. The most studied antibiotics belong to the tetracycline and penicillin classes, with oxytetracycline being most frequently investigated, followed by penicillin, tetracycline, ampicillin, chlortetracycline, cloxacillin, and amoxicillin. The findings confirm that no single strategy is universally effective. The results indicate that the future of ARs mitigation lies in the development of synergistic and combined approaches to achieve industrially viable, cost‐effective protocols that preserve product quality. This study highlights the need for future research focused on selective technologies and the assessment of by‐product toxicity, alongside the regulatory aspects for the application of these technologies.

## 1 Introduction

The use of antimicrobials, particularly antibiotics, in dairy production is a widespread practice for the treatment of clinically ill animals (such as those with mastitis and other infections) (Sachi et al. 2019; Smithers 2023) or to prevent the occurrence of diseases, to promote growth, and to improve feed efficiency (Akinyemi et al. 2021; Martin 2011; Jeena et al. 2020). However, the indiscriminate, inappropriate, or excessive use of antibiotics leads to the presence of antibiotic residues (ARs) in animal‐derived foods (Piñeiro and Cerniglia 2021), including milk, posing a significant challenge to public health and the dairy industry (Aning et al. 2007; Roca et al. 2011; Akinyemi et al. 2021). Exposure to ARs through the consumption of dairy products is associated with human health risks, including allergic reactions, disruption of the normal intestinal flora, bone marrow aplasia, and the transfer of resistant bacterial strains (Brown et al. 2020; Virto et al. 2022; Beyene 2015; Bacanlı 2024). Furthermore, the presence of ARs in milk can compromise industrial processes, such as the fermentation of yogurt and cheese by inhibiting the starter cultures used (László et al. 2018; Layada et al. 2016; Mohamed et al. 2020; Mangsi et al. 2021), leading to economic losses (Priyanka et al. 2017; Smithers 2023; Beltrán et al. 2018).

In developing countries, approximately 0.1%–0.5% of tanker milk samples test positively for ARs (Smithers 2023). Thus, the growing interest of the dairy industry in reducing or inhibiting ARs activity in milk has prompted significant research, and numerous studies have been conducted on techniques and strategies to mitigate these residues (Omairi et al. 2022). The primary strategy for controlling ARs is preventing them at the source, through strict Good Veterinary Practices (GVP) such as the concise use of antibiotics and adherence to withdrawal periods. However, failures in these systems still occur, thus necessitating alternative mitigation strategies (Varga and Szigeti 2016).

Mitigation technologies have been the subject of intensive research. Several studies have shown that heat treatment reduces ARs in milk (Han et al. 2015; Rana et al. 2019; Zorraquino et al. 2009, 2011; Roca et al. 2011; Junza et al. 2014), but their effectiveness is variable and there is a negative impact on the nutritional quality (reduction of micronutrients) and may alter the sensory properties of milk due to the application of high temperatures (J. Chen et al. 2024). Given this, emerging nonthermal technologies such as pulsed electric field (PEF), high pressure, supercritical carbon dioxide, ultraviolet (UV) light, cold plasma (CP), ozone, and pulsed light (PL) have appeared as promising alternatives with potential to degrade residues while preserving product quality (Neoκleous et al. 2022; J. Chen et al. 2024; Allai et al. 2023). Other approaches, including biological (fermentation) and adsorption methods, are also being explored. However, reports on applying these technologies to reduce or inhibit ARs in milk are still scarce (Shinde et al. 2021, 2022; Alsager et al. 2018; Liu et al. 2022).

Despite the growth of research, a gap persists in the literature for a systematic, critical analysis that consolidates current knowledge, compares the efficacy of different technologies, and evaluates their industrial feasibility and regulatory context. The landscape of the research is fragmented, and a clear overview of the trends, key players, and future directions in this field is lacking. Therefore, this study aims to fill this gap through a scientometric and systematic review of the literature on strategies used to mitigate ARs in milk and dairy products. By mapping the evolution of research, identifying the most promising technologies, and critically analyzing knowledge gaps, this review aims to inform future research directions and support policy and industry practices.

## 2 Methodology

The data collection and analysis for this systematic review were conducted between October 2024 and April 2025, adhering to the guidelines of the Preferred Reporting Items for Systematic Reviews and Meta‐Analysis (PRISMA) (Page et al. 2021) and the bibliometric analysis (Donthu et al. 2021). To minimize selection bias, the searches were conducted independently by two reviewers, Emelda Simbine Ribisse (ESR) and Wilma Custódio Fumo (WCF). The research team developed a predefined protocol outlining the search strategy, inclusion and exclusion criteria, and metadata extraction process. Study quality was assessed using an adapted version of the Joanna Briggs Institute (JBI) Critical Appraisal Checklist (Aromataris et al. 2015).

### 2.1 Search Strategy and Selection Criteria

Published literature related to strategies for reducing ARs in milk and dairy products was collected from six electronic databases: Web of Science, Scopus, PubMed, Embase, AGRICOLA, and CAS Abstract. The search was performed using combinations of the keywords such as “Milk” AND (“*dairy products*” OR “*milk production*”) AND (“*antibiotic residues*” OR “*antimicrobial residue*”) AND (“*antibiotic reduction*” OR “*antibiotic removal*”). In the database search strategy, bibliographic review articles and book chapters were excluded. In addition, other relevant studies omitted from electronic databases were manually screened as secondary sources.

**FIGURE 1:**
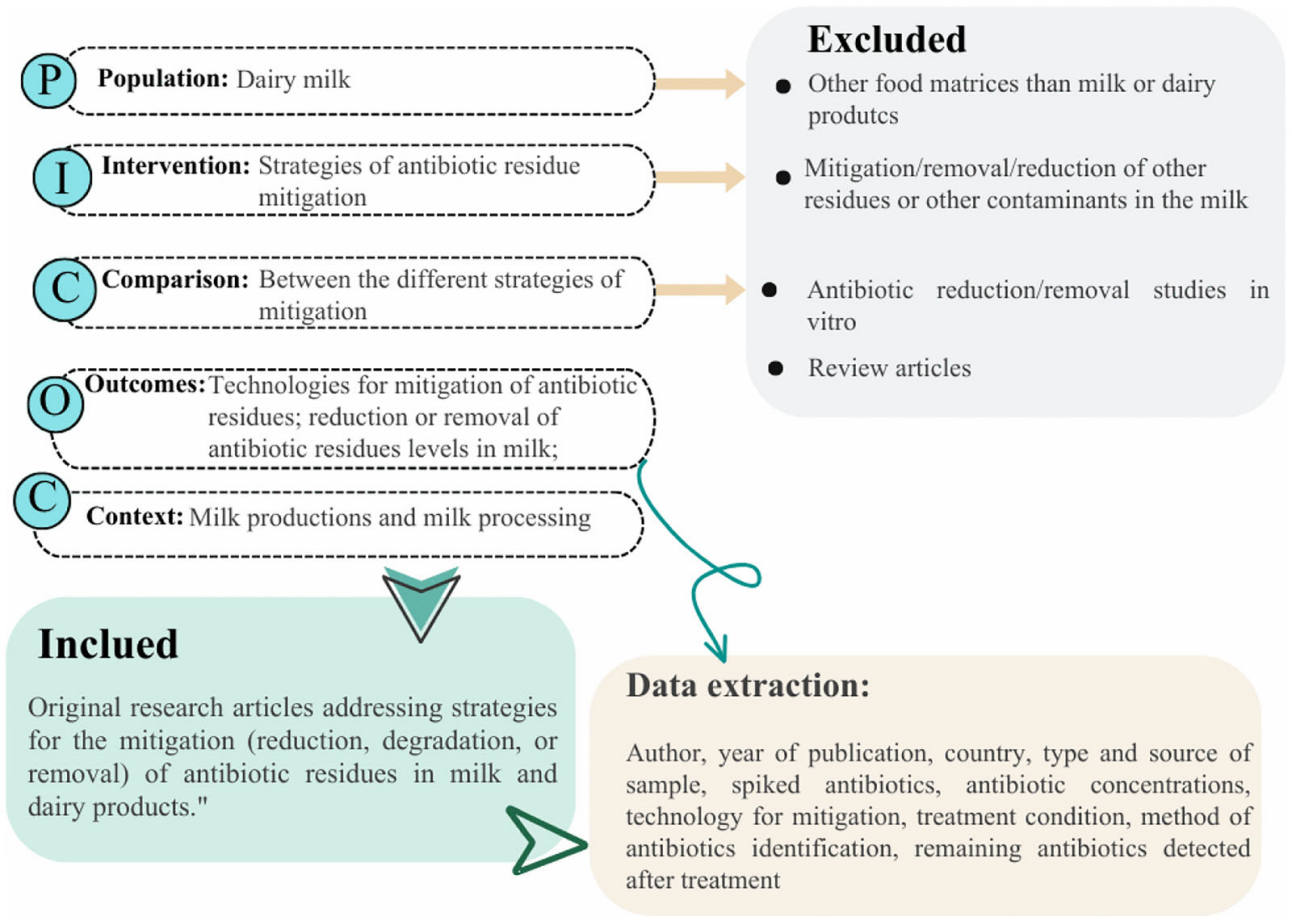
Schematic representation of the PICOC framework applied in this systematic review.

The study selection was guided by the PICOC framework (Population, Intervention, Comparison, Outcome, and Context). A visual summary of the PICOC components, the eligibility criteria used, and the main elements extracted from each selected study is shown in Figure 1. The population of interest was dairy milk; thus, all original articles focusing on ARs in milk and processed milk were included, while studies that did not address dairy products were excluded. The intervention considered was the mitigation of ARs in milk, focusing on the main methodologies employed for their reduction or removal. Studies addressing the mitigation of other residues or contaminants unrelated to antibiotics were excluded. Comparisons were made among different ARs mitigation strategies, with a particular focus on thermal, nonthermal, and biological technologies. Outcomes included data on the effectiveness of these technologies in reducing or eliminating ARs in milk. The context of the studies was limited to milk production and milk processing.

### 2.2 The Survey Process

**FIGURE 2:**
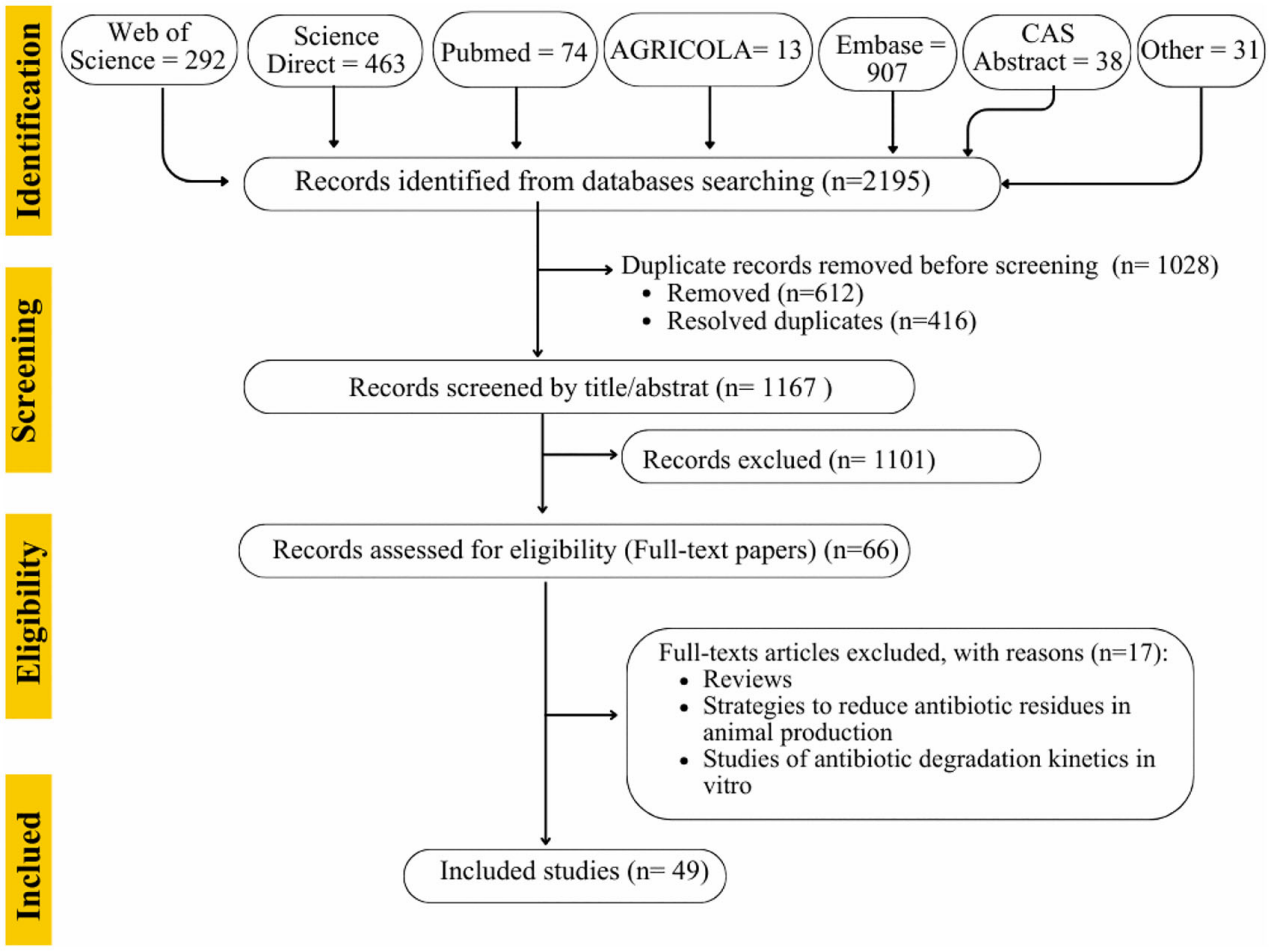
PRISMA flow diagram outlining the identification, screening, eligibility assessment, and inclusion process of studies for this systematic review.

The selection process is summarized in the PRISMA flow diagram (Figure 2). A total of 860 published articles were retrieved from Web of Science (*n* = 292), Science Direct (*n* = 463), PubMed (*n* = 74), and other sources (*n* = 31). All articles retrieved from databases and manually sourced were imported into the literature review software Rayyan (Ouzzani et al. 2016) for the selection process. After the initial assessment, 15 duplicate records marked as ineligible by automation tools (8 deleted and 7 resolved) were removed. A total of 845 studies remained for screening. The articles were selected by two reviewers (E.S.R. and W.C.F.), first by screening records based on title and abstract. The articles were unanimously selected and evaluated for eligibility. Based on the screening of the title and abstract, 790 records were excluded because they did not meet the inclusion criteria. The full articles selected were managed in PDF format using the reference manager Mendeley. After assessing the eligibility of 55 full‐text articles, 14 were excluded for the following reasons: review articles, studies focused on strategies to reduce ARs in animal production, and in vitro studies on antibiotic degradation kinetics. Then, 49 original research articles were included in the present systematic review.

### 2.3 Quality Assessment

The JBI Critical Appraisal Checklist (Aromataris et al. 2015) was adapted and used as a tool to assess the methodological quality of the risk‐of‐bias assessment in the included studies. This adapted tool consists of nine criteria that evaluate aspects such as the clarity of inclusion criteria, comparability of experimental conditions, detailed description of mitigation strategies, validity of analytical methods for residue detection, completeness of sample follow‐up, adequacy of statistical analyses, and alignment between results and conclusions.

Two reviewers (E.S.R. and W.C.F.) conducted the quality assessment independently, and disagreements were resolved through consensus. A final, agreed‐upon rating was assigned to each study. Each criterion was rated as Yes, No, Unclear, or Not Applicable, and the included studies were scored based on the number of “Yes” responses. The studies were classified as “high quality” (8–9 “Yes”), “moderate quality” (6–7 “Yes”), “low quality” (4–5 “Yes”), and “very low quality” (0–3 “Yes”).

**FIGURE 3:**
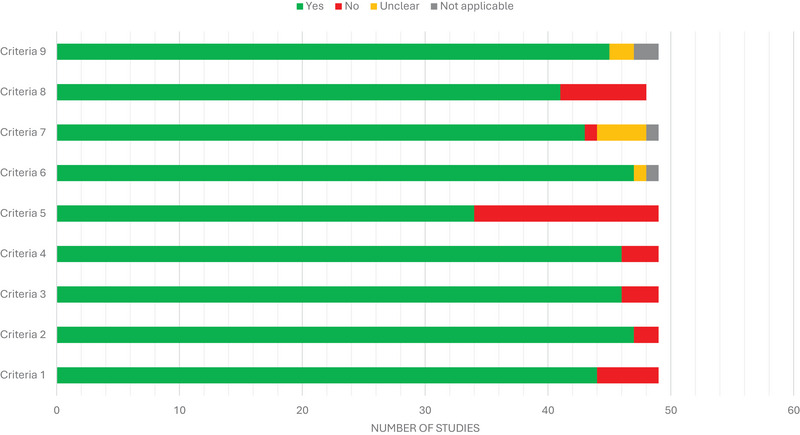
Summary bar chart of the methodological quality assessment for each criterion evaluated in the studies included in the systematic review. Each bar represents one criterion, with color‐coded segments indicating the number of studies receiving “yes” (green), "no” (red), “not applicable” (gray), or “unclear” (yellow) assessments.

Among the 49 included studies, 79.6% (39/49) were classified as “high quality,” 16.3% (8/49) as “moderate quality,” and only 4.1% (2/49) as “low quality” (Figure 3). This distribution underscores the overall rigor and transparency of the selected articles, reflecting the use of well‐documented mitigation protocols and comprehensive sample follow‐up. Criterion 5 (validity and reliability of outcome measures) shows the highest proportion of negative assessments (30.6%), while Criterion 2 and 6 (complete follow‐up of samples) demonstrate the strongest performance with 95.9% positive assessments. The overall pattern reveals high methodological quality across most criteria, while also identifying specific areas for improvement in future research.

### 2.4 Scientometric Analysis

The scientometric analysis of the included studies on ARs mitigation strategies in milk was carried out using two complementary tools: the R package Bibliometrix (Aria and Cuccurullo 2017) and VOSviewer (version 1.6.20). Through Bibliometrix, various bibliometric parameters were assessed, including the temporal distribution of publications, the spatial distribution of scientific output, the countries with the highest publication counts, author productivity over time, Lotka's law, and the principal institutional affiliations. In VOSviewer, author collaboration networks and keyword co‐occurrence were analyzed. All results were compiled and presented as tables or illustrative charts to facilitate data visualization and interpretation.

## 3 Results and Discussion

### 3.1 Scientometric Analysis

#### 3.1.1 Temporal and Geographical Distribution of Publications About ARs Mitigation Research

**FIGURE 4:**
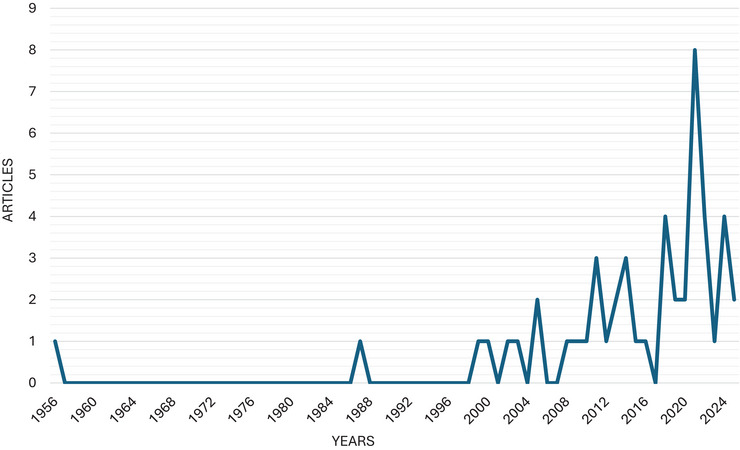
Temporal evolution of scientific publications on strategies for antibiotic residue mitigation in dairy products from 1956 to 2025.

The temporal evolution of scientific publications addressing strategies for the mitigation of ARs in dairy products is shown in Figure 4. The scientific production was categorized into three distinct phases: initial (I), development (II), and growth (III).

In Phase I, scientific production was incipient, with only two isolated publications‐one in 1956 and another in 1986‐representing 2.0% (2/49) of the total records. This suggests that while the topic was already recognized earlier, it had been around for decades but had not yet attracted consistent, sustained research attention. Phase II, spanning from 1999 to 2010, marked a period of gradual growth and increased regularity in publication frequency. During this period, the publication remained relatively stable, with approximately one article published each year. A total of nine articles were published (18.4% of the total) in this period, reflecting the emergence of more structured scientific efforts and a growing awareness of the importance of monitoring and mitigating ARs in dairy products. Phase III, spanning 2011–2024, represents a period of rapid growth, marked by a significant increase in scientific production output. This period accounts for the majority of publications, approximately 77.6% (38/49), with notable peaks in specific years, particularly in 2021, which alone contributed 16.3% (8/49) of total publications. This trend data suggests a growing interest in mitigating ARs in milk and dairy products, likely driven by the increasing global concern about their impact on public health.

**FIGURE 5:**
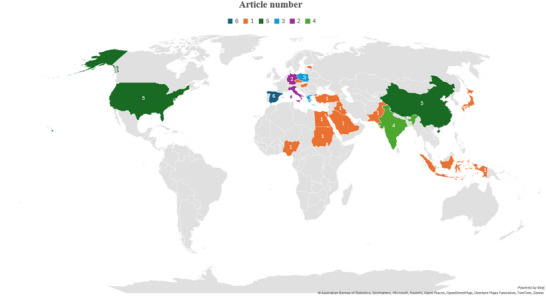
Geographical distribution of scientific publications on mitigation strategies for antibiotic residues in dairy products. The map illustrates each country's relative contribution, expressed as the total number of publications.

The geographical distribution of studies addressing mitigation strategies for ARs in dairy products is illustrated in Figure 5. The findings suggest that this is a topic of global scientific interest, though notable disparities exist in research contributions across continents and countries. The studies analyzed originated from four continents: Asia, with the highest diversity of contributing countries (China, India, Iran, Turkey, Iraq, Pakistan, and Japan); Europe (Spain, Italy, Greece, and Germany); Africa (Nigeria and Egypt); and the Americas (the USA). Among the 49 articles analyzed, a total of 22 countries were identified as contributors to the scientific literature on mitigation strategies for ARs in dairy products.

#### 3.1.2 Correlation Between Research Output and Milk Production

The eight most productive countries account for approximately 44.9% (22/49) of the total scientific production on this topic, as shown in Table 1. Spain emerged as the leading contributor, accounting for approximately 12.2% (6/49) of the publications. Although its first recorded publication dates back to 2008, its subsequent output has placed the country at the forefront of research in this field. Soon after, the United States (USA) and China followed with 10.2% (5/49). India ranks third with 8.2% (4/49), followed by Poland and Greece, each with 6.1% (3/49), Italy and Germany with 4.1% (2/49). Several other countries, including Egypt, Turkey, Nigeria, Pakistan and Iran, contributed with one publication each.

**TABLE 1:** Comparative analysis of the top eight contributing countries to research on antibiotic residue mitigation strategies in milk.

Countries	Rank of publication	Publications number	Percentage of contribution (%)	First published year	Rank of milk production^a^	Milk production (t)^b^
Spain	1	6	12.20	2008	21	7 564 500
USA	2	5	10.20	1997	2	102 677 105
China	3	5	10.20	2016	4	42 431 560
India	4	4	8.20	2019	1	127 105 140
Greece	5	3	6.10	2022	59	669 620
Poland	6	3	6.10	2013	12	15 482 240
Italy	7	2	4.10	2018	14	13 058 970
Germany	8	2	4.10	2003	6	34 012 620

^a^ World Milk Production Ranking, 2023 (FAO 2024).

^b^ Global Milk Production by Country in 2023 (in metric tons) (FAO 2025).

**FIGURE 6:**
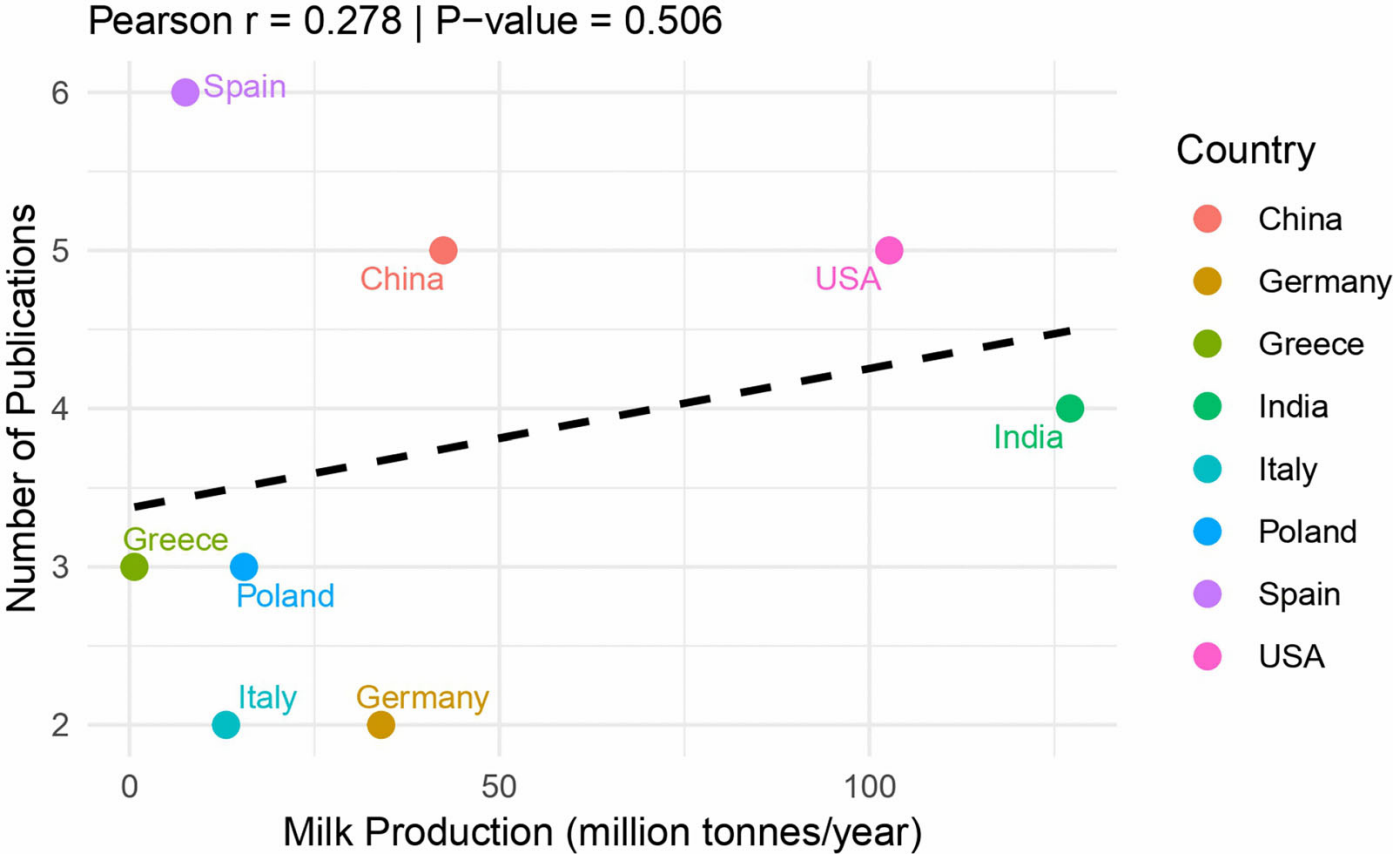
Scatter plot showing the relationship between annual milk production (million tons) and the number of publications on ARs mitigation strategies for eight countries. Each point represents a country, with Spain showing the highest publication count (6) despite low production (8.5 million tons), while India shows a moderate publication count (4) despite being the world's largest producer (213.8 million tons). The linear trend line (dashed black) indicates a weak, non‐significant positive correlation (*r* = 0.278, *p* = 0.506).

To contextualize the research output, the correlation between the number of publications and the total milk production of the top contributing countries was analyzed (Table 1 and Figure 6). Notably, there is no statistically significant correlation (Pearson *r* = 0.278, *p* = 0.506). Spain, a country with relatively low total milk production (8.5 million tons, ranking 21st globally, FAO 2025), leads in research output, with six publications. Conversely, India, the world's largest milk producer (with 213.8 million tons), contributed only four publications. This pattern suggests that national research priorities, regulatory frameworks, and funding availability may be stronger drivers of research in this area than production volume alone. The concentration of research in European countries, despite their moderate production levels, likely reflects the stringent regulatory environment of the European Union regarding ARs in food products.

#### 3.1.3 Leading Author, Productivity, and Collaboration Network

**FIGURE 7:**
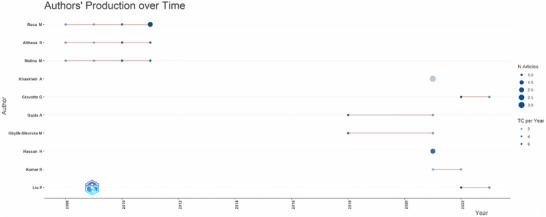
Temporal distribution of scientific production and citation impact of key authors in the field from 2008 to 2023. The figure illustrates the publication patterns of 10 researchers over 15 years. Each horizontal line represents an author's active publication timeline, with circle size proportional to the number of articles published (N. Articles, ranging from 1.00 to 2.00) and different symbols indicating citation impact (TC per Year, ranging from 1 to 5).

The scientific output of the ten leading authors in this thematic area is illustrated in Figure 7, where three distinct temporal patterns of publication can be observed. The earliest pioneers—Molina, Ricci, and Althaus—published primarily between 2008 and 2012, and they show the most significant overall impact. Many other authors exhibit a low to moderate volume of publications, as indicated by smaller to medium‐sized circles. Notably, there is an 8‐year gap (2012–2020) during which none of these authors published on this topic. This hiatus may reflect a temporary stagnation in the field, a generational shift in methodology that allowed new investigators to assume leadership, or a temporary lack of funding or interest in the field. Subsequently, the latest trends and methods emerged, and several new authors began publishing on the subject. Similar temporal patterns among specific authors suggest the existence of collaborative networks. Accordingly, a co‐authorship analysis was conducted to investigate these collaborations further.

**FIGURE 8:**
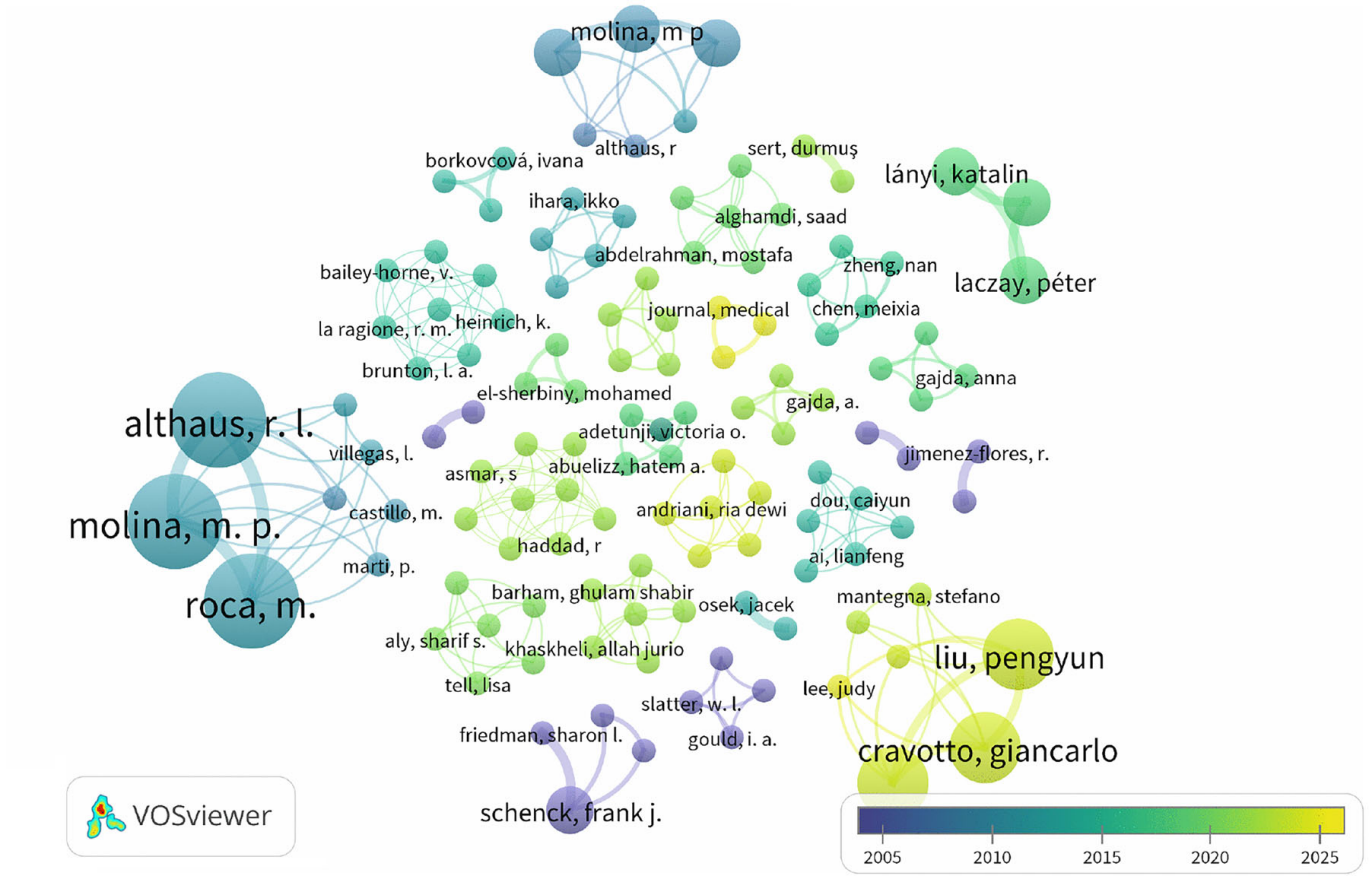
Co‐authorship network map visualizing collaborations between researchers, generated with VOSviewer software. Each circle represents an author, and the size of the circle is proportional to their productivity (measured by the number of publications) or impact within the analyzed dataset. The lines indicate collaborations in publications. The colors of the nodes and the timeline (approximately 2005–2025) illustrate the average publication period of the authors.

Regarding co‐authorship, Figure 8 illustrates the network of scientific collaboration among authors. Collaboration clusters are visualized based on color, indicating both the connections among researchers and the average publication period. This analysis enables the identification of the most prolific and influential authors, the leading research groups, and their respective temporal dynamics of productivity.

Four main collaboration clusters stand out: (1) the light blue cluster (located on the left), which gathers central collaborators who interact frequently in this research area. Authors such as R.L. Althaus, M.P. Molina, and M. Roca, are prominent in this group, characterized by a significant number of publications or impact, with their publication activity peaking in the mid‐range period of the analysis (approximately 2008–2015). (2) The light green cluster highlights a broader and slightly more dispersed group, whose publication activity appears to be concentrated in a more recent period (e.g., between 2018 and 2022). (3) The yellow cluster (located in the lower right portion of the map) represents a set of researchers with more recent activity, approximately between 2020 and 2025. This group is the second most prominent in terms of authors investigating the topic, with Liu, Pengyun and Cravotto, Giancarlo standing out as leading figures and (4) the purple cluster, predominantly at the bottom of the network, with some more scattered connections, includes authors with earlier publications (approximately 2005–2010). Researchers such as F.J. Schenck, S.L. Friedman, W.L. Slatter, I.A. Gould, and R. Jimenez‐Flores are representatives of this group. They can be considered pioneers in the study of this topic. The configuration of the clusters suggests that research on strategies for mitigating ARs in milk is characterized by the activity of multiple collaboration groups, possibly with different geographical or thematic focuses. The centrality of certain authors within their respective clusters, indicated by larger nodes and a higher number of connections, reinforces their position as key researchers or leaders of research groups in the field.

**FIGURE 9:**
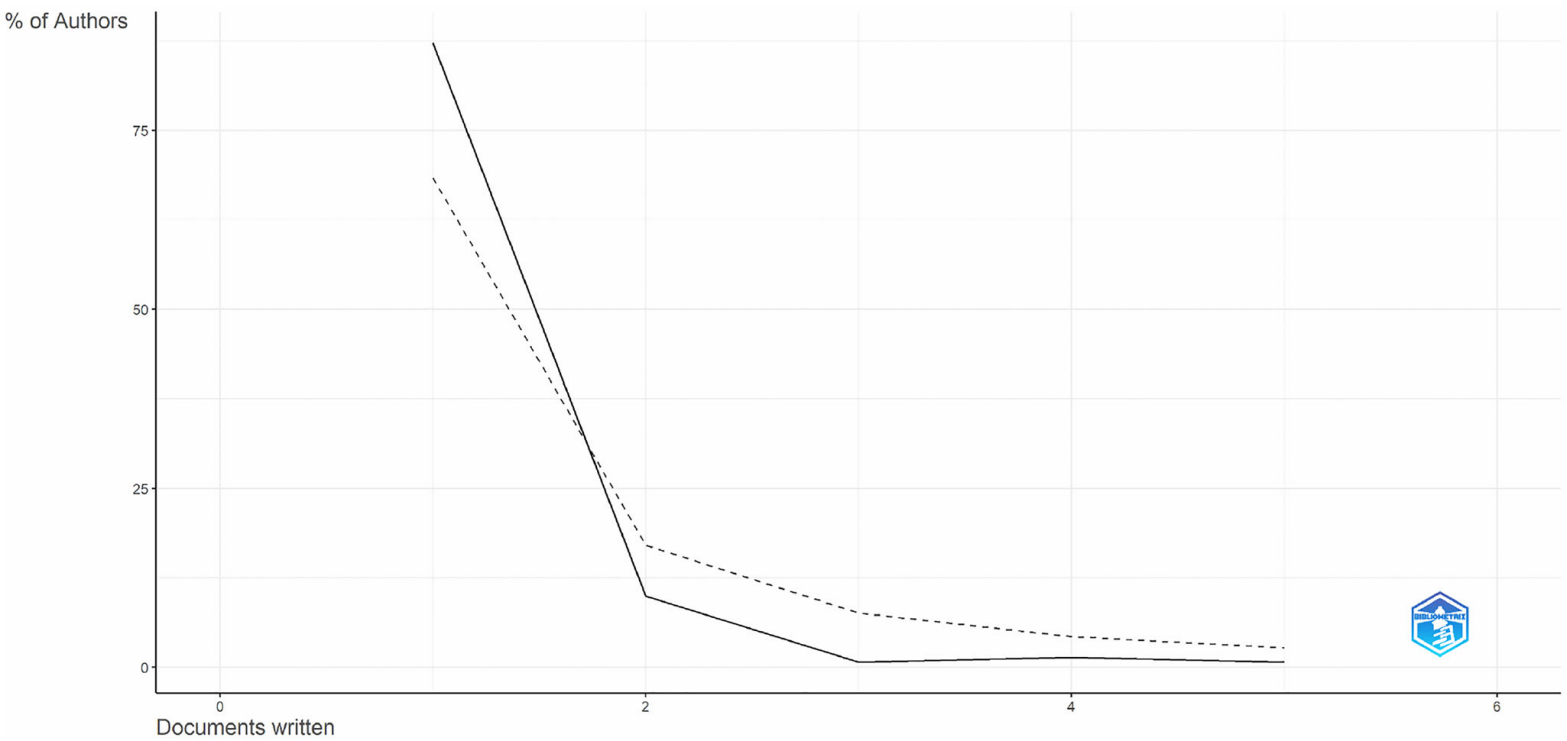
Distribution of author productivity according to Lotka's law. The *Y*‐axis represents the percentage of authors, and the *X*‐axis shows the number of published documents. The solid line indicates the observed distribution in the analyzed dataset, while the dashed line represents the theoretical distribution expected by Lotka's law. The graph shows a predominance of authors with a single publication and a sharp decline in the percentage of authors with multiple publications.

The author's productivity in the field of strategies for mitigating ARs in milk was analyzed using Lotka's law. According to Figueiredo et al. (2019), Lotka's law is a bibliometric principle that describes the frequency of publications by authors. The results, illustrated in Figure 9, reveal that author productivity in this study (solid line) follows the general pattern of Lotka's law (dashed line). It is observed that most researchers (approximately 90%) contributed only one publication, while a small number of authors (less than 5%) were responsible for multiple publications. This pattern indicates that research in this field is predominantly driven by a core group of more prolific authors, supported by a broader base of researchers who contribute more sporadically or focus on a single study. In the classical Lotka distribution, it is expected that approximately 70%–75% of authors contribute only one work, and about 20%–25% contribute two or more works.

A slight discrepancy between the observed curve (solid line) and Lotka's theoretical curve (dashed line) can be noted. Specifically, the sharper decline in the observed curve for authors with two or more publications suggests that this particular research field may have a particularly high proportion of “one‐time contributors.” Alternatively, this may indicate that the exponent of Lotka's law that best fits this dataset differs from the classical value of 2.

#### 3.1.4 Co‐Occurrence Keywords and Thematic Evolution

**FIGURE 10:**
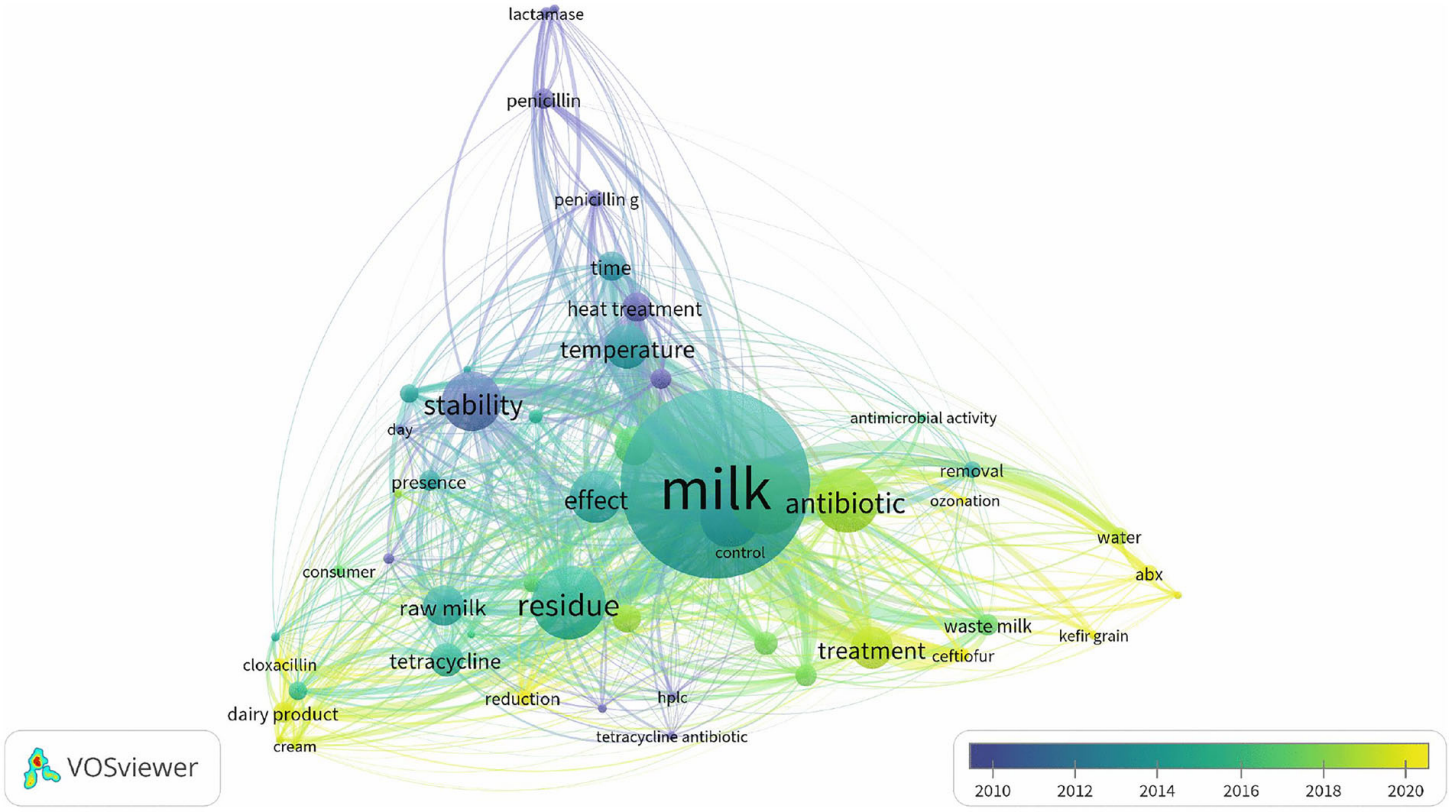
Network map of keyword co‐occurrence related to mitigation of antibiotic residues in milk, generated using VOSviewer software. Each circle represents a keyword, and its size indicates the frequency or relevance of the term in the analyzed articles. The lines connecting the circles show that these terms frequently appear together. Thicker or shorter lines may indicate a stronger relationship. The terms are grouped by color, forming “clusters.” Terms within the same cluster tend to be more closely related, representing specific subtopics or research areas. The color bar at the bottom suggests a temporal analysis (2010–2020), where the color of each term indicates the average period in which it was most prominent in publications.

The results of the co‐occurrence analysis of keywords extracted from the scientific articles are illustrated in Figure 10. The term “milk” appears as the largest and most central node in the map, indicating it is the focus of research in this field. The greenish‐blue color of this term suggests that it has been a consistent topic of interest throughout the analyzed period, with emphasis between 2014 and 2016, according to the timeline. Other frequently occurring terms include “antibiotics,” “residue,” “stability,” and “treatment.” A total of 52 keywords were observed, organized into three main clusters, where the colors represent thematic groupings and the average year of publication associated with each term.

The first cluster, shown in purple–blue (2010–2014), represents the oldest studies. It includes terms such as *penicillin*, *lactamase*, *time*, and *temperature*, suggesting that during this period, the research focus was mainly on the enzymatic and thermal degradation of antibiotics, particularly β‐lactams like penicillins. The second cluster, ranging from light blue to green (2014–2018), reflects an intermediate research phase. It includes terms such as *residue*, *raw milk*, *stability*, *storage*, *consumer*, and *dairy products*. This cluster highlights growing concern over the presence and stability of ARs in raw milk and dairy products, as well as the implications for food safety and consumer health. The third cluster, represented in light green to yellow (2018–2025), corresponds to more recent research. Prominent terms include *treatment*, *removal*, *ozonation*, *waste milk*, *antimicrobial activity*, *ceftiofur*, and *oxytetracycline* (OTC), reflecting a current focus on active strategies for mitigating ARs. These include efforts aimed at removal, degradation, and reduction of residues, with emphasis on emerging technologies such as ozonation and the reuse of waste milk.

In summary, the temporal evolution of keyword co‐occurrence reveals a thematic shift: from an initial focus on residue stability through thermal and enzymatic processes, to concerns about food safety and public health, culminating in recent studies that target mitigation strategies. Emerging, green, and nonthermal technologies—such as ozone, ultrasound, PEFs, and fermentative alternatives (such as *kefir* and lactic acid bacteria [LAB])—are current and promising trends for reducing ARs in milk and dairy products.

### 3.2 Overview of Strategies for ARs Mitigation in Foods

**FIGURE 11:**
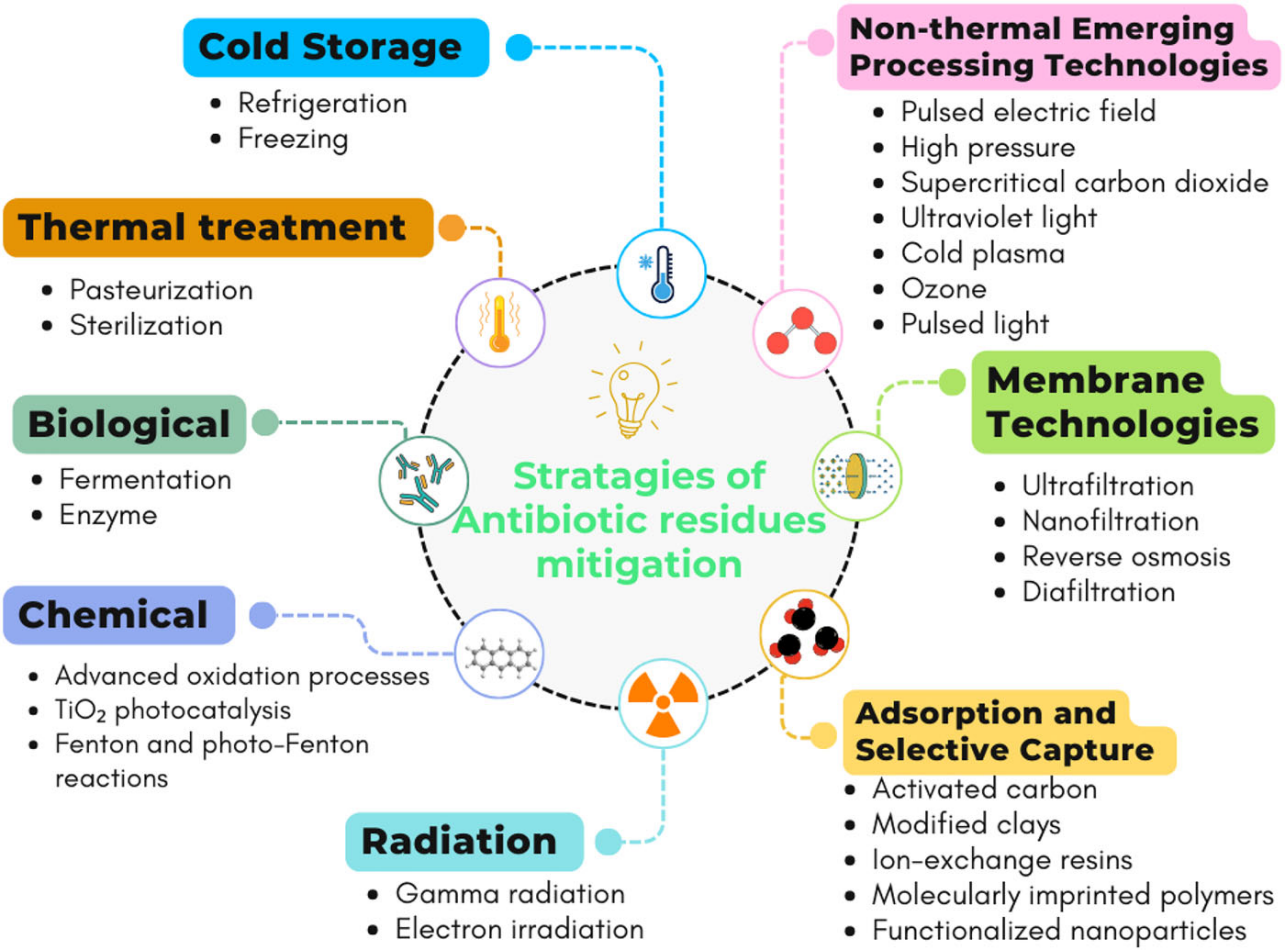
Overview of main techniques used in the mitigation of antibiotic residues.

The stability of ARs can be affected by various conditions and factors, leading to their degradation and loss of activity (Roca et al. 2011). Thus, various strategies can be employed to mitigate (reduce or eliminate) these residues (Figure 11). Thermal treatment represents the oldest and most traditional approach, widely used in the food industry, including the dairy sector (Roca et al. 2011; Zorraquino et al. 2011, 2008). Concurrently, biological methods, although also long‐established, are considered more sustainable and natural. An additional advantage lies in the potential utilization of residual raw materials for the development of new products (Grunwald and Petz 2003).

Advanced oxidation processes (AOPs) constitute highly efficient chemical approaches for decomposing pharmaceutical molecules, including antibiotics (Deng et al. 2019). These processes employ highly reactive chemical species, notably hydroxyl radicals (•OH), to degrade organic contaminants. Among them, heterogeneous photocatalysis with titanium dioxide (TiO_2_), which combines UV radiation with a semiconductor catalyst, demonstrates effectiveness in degrading multiple classes of antibiotics by generating potent oxidizing species (Deng et al. 2019; Kitazono et al. 2012). Applying radiant energy (ionizing or non‐ionizing radiation [IOR]) has also been proven effective in degrading organic compounds, including antibiotics (Chu et al. 2018; Tegze et al. 2019).

In addition, membrane technologies, based on the separation and removal of particles according to molecular size, electrical charge, and other physicochemical properties, are employed to retain ARs (Kosikowski & Jimenez‐Flores 1985). Adsorption techniques stand out for their high efficiency, low cost, and operational simplicity (Liu et al. 2024). They ensure the transfer of ARs from the aqueous phase to a solid adsorbent phase. Materials such as activated carbon, modified clays, and various nanoparticles have been used as effective adsorbents for different classes of antibiotics. More recently, emerging nonthermal technologies such as CP (Magureanu et al. 2011), ultrasound (Su et al. 2012), ozonation (Arslan‐Alaton and Caglayan 2006), and PEF (Shinde et al. 2022) have been gaining prominence in the mitigation of ARs and showing promising results.

### 3.3 Systematic Review Findings: Characteristics of the Included Studies of Strategies of ARs Mitigation in Dairy Products

The following sections provide a comprehensive analysis of the metadata extracted from the 49 articles included in this review on strategies for the mitigation of ARs in milk. The characteristics of the included studies are shown in Table 2.

**TABLE 2:** Characteristics of the included studies (*n* = 49) in the systematic review on ARs mitigation strategies in milk. The table details sample types, sample origin, antibiotics studied, classification of mitigation approaches (classic, emerging, or combined), specific strategies employed, and corresponding references.

Study no.	Sample	Sample origin	Antibiotic studied	Mitigation strategies				References
				Classic technology?	Emergency technology?	Combined methods?	Strategies of mitigation	
1	Whole raw milk	Veterinary farm	PEN G	Yes	No	Yes	Membrane technologies: Ultrafiltration and permeate washes	Kosikowski and Jimenez‐Flores (1985)
2	Unpasteurized and UHT milk	Bulk tank of a dairy farm	CEFQ	Yes	No	Yes	Heat treatment, pH adjustment, fermentation, and enzymatic treatment	Horton et al. (2015)
3	Raw milk	Bulk milk tank from a local farm	CLO	Yes	No	Yes	Heat treatment, fermentation (yogurt production), and freezing	Gbylik‐Sikorska et al. (2021)
4	Whole raw milk	Farm	OTC, TC, CTC, and DC	Yes	No	No	Heat treatment and processing (cheese production)	Gajda et al. (2018)
5	Saleable pasteurized homogenized whole milk	NI	CEF	Yes	No	Yes	Heat treatment and pH treatment	Garzon et al. (2020)
6	Raw cow milk, whole milk, and fat‐free milk	Store, located dairy farm	OTC, CTC, and TC	No	Yes	Yes	Electrochemical oxidation	Kitazono et al. (2012)
7	Skim milk powder	NI	AMO, AMP, CLO, PEN G, CEX, CEL, CEP, CEPI, and CEXM	Yes	No	No	Heat treatment	Roca et al. (2011)
8	Raw milk	Local farm	CIP, DAN, ENR, SAR, DIF, and FLU	Yes	No	No	Refrigeration and freezing	Chen et al. (2016)
9	Raw milk	Dairy farm	TC	yes	No	Yes	Heat treatment and pH treatment	Kurjogi et al. (2019)
10	Commercial UHT milk	NI	CIP, ENR, NOR, FLU, and OXA	Yes	No	No	Heat treatment	Roca et al. (2010)
11	Skim milk powder	NI	SDZ, STZ, SPD, SMR, SMTZ, SCL, SMX, and SQX	Yes	No	No	Heat treatment	Roca et al. (2013)
12	Raw milk	Dairy company	OTC and TYL	Yes	No	Yes	Heat treatment, acidification/heating	Hassan et al. (2021)
13	Raw milk	Local market	PEN‐G	Yes	Yes	Yes	Heat treatment and pulsed electric field	Shinde et al. (2021)
14	Skim milk	Dairy company	β‐Lactam and Tetracyclines	No	Yes	No	Ozone	Sert and Mercan (2021)
15	Pasteurized milk	Local milk suppliers	CEF, SMM, MAR, and OTC	No	Yes	Yes	Sonication; sonocatalytic, and activated carbon	Liu et al. (2024)
16	Raw milk	Local farm	AMO, DC, CIP, and SDZ	No	Yes	No	Ozone	Alsager et al. (2018)
17	Raw milk	Local market	PEN‐G, CLO, AMP, CEP, CEX, STM, NEO, SDZ, STZ, TC, and TRIM	Yes	No	No	Heat treatment—Boiling	László et al. (2018)
18	UHT milk	Local branch of two major supermarkets	CEF, SMM, MAR, and OTC	No	Yes	Yes	Sonolytic, addition of H_2_O_2_	Liu et al. (2023)
19	Pasteurized milk	Local branch of two major supermarkets	CEF, SMM, MAR, and OTC	No	Yes	No	Ozonation	Liu et al. (2022)
20	NI	NI	PEN G, AMP, AMO, CLO, CEP, CEFQ, CEXM, CEX e CEL	Yes	No	No	Heat treatment	Zorraquino et al. (2008)
21	Whole and skim milk	NI	PEN	Yes	No	Yes	Enzymatic treatment	Lee and Richardson (1987)
22	NI	NI	GEN; CAN, NEO; and STM	Yes	No	No	Heat treatment	Zorraquino et al. (2009)
23	Raw milk	Local farmer	AMO	Yes	No	No	Fermentation	Umam et al. (2023)
24	Raw milk	Vending machine	TC and OTC	Yes	No	No	Heat treatment	Kellnerová et al. (2014)
25	Raw skim milk	Dairy plant	PEN G	No	Yes	Yes	Chemical treatment, heat treatment, and fermentation	Hanway et al. (2005)
26	UHT milk	NI	ERY, SPI, TLY and LIN	Yes	No	No	Heat treatment	Zorraquino et al. (2011)
27	Raw milk	Farmer	OTC	Yes	No	No	Heat treatment (thermisation)	Cabizza et al. (2018)
28	NI	Local markets	PEN G, OTC, GEN and sulfonamide	Yes	No	No	Heat treatment	Khaskheli et al. (2021)
29	NI	NI	PEN G, PEN V, and AMP	Yes	No	No	Enzymatic treatment	Li et al. (2014)
30	Raw milk	NI	PEN G, AMP, CLO, CEF and OTC	Yes	No	No	Refrigeration and freezing	Rózańska and Osek (2013)
31	Raw milk	Local markets	NI	Yes	No	No	Heat treatment	Almashhadany (2021)
32	Whole milk	Local milk distributors	β‐Lactam, Tetraciclynes and sulfonamide	No	Yes	No	Adsorbent method (biochar): Prosopis wood, coconut shell, and rice husks	Suguna Devakumari (2021)
33	Raw milk	Farmer	CTC, DEM, METC, MINO, OTC, and TC	Yes	No	No	Refrigeration	Podhorniak et al. (1999)
34	Raw milk	Localities	OTC	Yes	No	No	Heat treatment	Faried et al. (2024)
35	Raw milk, cheese, and yoghurt	Dairy company	PEN G, STM, and TC	Yes	No	No	Heat treatment, fermentation	Adetunji (2011)
36	Raw milk	NI	PEN	Yes	No	No	Heat treatment and storage	Shahani et al. (1956)
37	Raw milk	Bulk tank	AMP	Yes	No	No	Refrigeration and freezing	Schenck and Friedman (2000)
38	Whole milk	Local market	SMTZ	Yes	Yes	Yes	Heat treatment and pulsed electric field	Shinde et al. (2022)
39	UHT milk	Markets	PEN, CLO, OXA, DIC, AMP, or NAF	Yes	No	No	Heat and fermentation	Grunwald and Petz (2003)
40	Raw milk	Small dairy farms, street peddlers and dairy shops	OTC and SMTZ	Yes	No	No	Heat treatment	Fathy et al. (2019)
41	Raw milk	Dairy farmer	OTC, TC, and CTC	Yes	No	No	Heat treatment	Loksuwan (2002)
42	Pasteurized milk	Commercially	PEN G	Yes	No	Yes	Heat treatment, acidification/heating, enzymatic	Nitz et al. (2005)
43	Raw milk	Animal husbandry	ENR	Yes	No	No	Heat treatment	Widiyanti et al. (2022)
44	Raw milk	NI	PEN G	Yes	No	No	Heat treatment	Sats et al. (2014)
45	Fresh milk	Farm	TYL, ENR, AMP and OTC	Yes	No	No	Heat treatment	Alawad et al. (2025)
46	Raw milk	Local market	TC	Yes	Yes	Yes	High hydrostatic pressure (HHP), heat treatment and ultrasound	Sidirokastritis et al. (2022)
47	Fresh skimmed milk	Local supermarket	OTC	No	Yes	No	Pulsed electric field (PEF)	Wen et al. (2025)
48	Milk and yogurt	Local supermarket	TC	No	Yes	No	Metal–organic frameworks (MOFs) with high porosity have	Li et al. (2020)
49	Milk and yogurt	NI	SMTZ	Yes	No	No	Pasteurizing, boiling, autoclaving and microwaving	Papapanagiotou et al. (2005)

Abbreviations: AMO, amoxicillin; AMP, ampicillin; CAN, canamicin; CEF, ceftiofur; CEFQ, cefquinome; CEL, cephalonium; CEP, cefoperazone; CEPI, cephapirin sodium salt; CEX, cephalexin; CEXM, cephuroxime; CIP, ciprofloxacin; CLO, cloxacillin; CTC, chlortetracycline; DAN, danofloxacin; DC, doxycycline; DEM, demeclocycline; DIC, dicloxacillin; DIF, difloxacin; ENR, enrofloxacin; ERY, erythromycin; FLU, flumequine; GEN, gentamicin; LIN, lincomycin; MAR, marbofloxacin; METC, methacycline; MINO, minocycline; NAF, nafcillin; NEO, neomycin; NI, not indicated; NOR, norfloxacin; OTC, oxytetracycline; OXA, oxolinic acid; PEN G, penicillin G; SAR, sarafloxacin; SCL, sulfachloropyridazine; SDZ, sulfadiazine; SMM, sulfamonomethoxine sodium; SMR, sulfamerazine; SMTZ, sulfamethazine; SMX, sulfadimethoxine; SPD, sulfapyridine (SPD); SQX, sulfaquinoxaline; STM, streptomycin; STZ, sulfathiazole; TC, tetracycline; TRIM, trimethoprim; TYL, tylosin.

Regarding the samples evaluated in the included studies, raw milk (including variations such as “whole raw milk” and “unpasteurized milk”) was the most frequently analyzed, accounting for approximately 67.3% (33/49) of the studies. Processed milk samples (pasteurized, ultrahigh temperature [UHT], skim milk, and skim milk powder) were evaluated in approximately 26.5% (13/49) of the studies. Only 8.16% (4/49) of the studies assessed dairy derivatives, such as yogurt and cheese. This predominance of raw milk samples suggests a focus on evaluating the presence of ARs directly at the source, namely, dairy cattle production, and on applying mitigation strategies before the industrial processing of this raw material. The evaluation of processed milk may be related to the persistence of ARs even after thermal treatments.

Regarding the origin of the samples, their diversity is directly related to the types of samples that are most frequently evaluated. Farms or milk tanks (considered primary sources) constituted the most predominant sample origin, accounting for approximately 36.7% (18/49) of the studies. Markets and points of sale accounted for approximately 32.7% (16/49) of the studies, while only 6.1% (3/49) evaluated samples from milk processing industries.

Our analysis of 49 studies identified 59 instances of AR mitigation strategies, which were categorized into seven different groups. Thermal treatment was by far the most prevalent, comprising 67.8% (40/59) of all reported methods. Within this category, conventional heat treatment, such as boiling, pasteurization, and sterilization, was most common, representing 80.0% (32/40) of all thermal methods discussed (Table 3). Following thermal methods, biological treatment and emerging nonthermal processing technologies, each representing 16.9% (10/59) of instances. Less frequently reported strategies included refrigeration and freezing techniques at 8.5% (5/59), chemical treatment at 6.8% (4/59), and adsorptive methods at 5.1% (3/59). Membrane technology was the least explored, with only a single reported instance (1.7%).

**TABLE 3:** Effect of thermal methods on reducing antibiotic residues in dairy milk.

Study no.	Concentration on target antibiotics	Conditions	Method for identification	Main Findings	References
2	2 µg/mL (CEFQ)	(i) Heat treatment: 4°C, 10°C, 37°C, and 50°C for up to 144 h	LC–MS/MS	Cefquinome declined steadily at 37°C, degradation (86%) was more rapid at 50°C; pH: cefquinome degradation and concentrations decreased below the limit of quantification (125 µg/kg) at pH 10 after 8 h	Horton et al. (2015)
		(ii) pH: 1 (197 ± 2 µL of 10 mol/L HCl), pH 4 (117 ± 1 µL of 5 mol/L HCl), or pH 10 (159 ± 1 µL of 2 mol/L NaOH)			
4	100 µg/kg for each antibiotic (OTC, TC, CTC, and DC)	63°C for 30 min	LC–MS/MS	Not significant decreases in the levels of TC's residues in milk: 10% for OTC, 11% for TC, 19% for CTC, and 6% for DC	Gajda et al. (2018)
5	200 and 400 ppb (CEF)	63°C for 30 min (LTLT), 72°C for 15 s (HTST) and 92°C for 20 min (HTLT)	HPLC; LC–MS/MS	Alkalinizing milk to pH 10 and heating milk to 92°C for 20 min degraded ceftiofur; 35.24% degradation of ceftiofur in HTLT	Garzon et al. (2020)
7	5000 µg/kg (AMO, AMP, CLO, PEN G, CEX, CEL, CEP, CEPI, and CEXM)	60°C, 70°C, 80°C, 90°C and 100°C for 0, 60, 120, 180, 240, 300, 360 min	HPLC	120°C for 20 min: degradation of amoxicillin (47.6%), ampicillin (84%), up to 90% for cephalosporins, except for cefquinome (79.9%), and losses of up to 100% for cefoperazone and cefuroxime. Degradation of β‐lactams not exceeding 1%	Roca et al. (2011)
9	1 mg/mL (TC)	70°C and 100°C for 24 h	HPLC	High temperatures of 70°C and 100°C affected the stability and subsequent antibacterial activity of azithromycin; a significant reduction in the effect of azithromycin and tetracycline by acid pH 4–5	Kurjogi et al. (2019)
10	1500 µg/kg (CIP, ENR, NOR, FLU, and OXA)	(i) 80°C for 0, 30, 60, 90, 120, 150, and 180 min	HPLC	Resistant to different temperatures; low degradation (< 6% in all cases); 12.71% (ciprofloxacin), 12% (norfloxacin) at 120°C/20 min	Roca et al. (2010)
		(ii) 100°C for 0, 30, 60, 90, 120, 150, and 180 min			
		(iii) 120°C for 0, 10, 20, 30, and 40 min			
11	200 µg/L (SDZ, STZ, SPD, SMR, SMTZ, SCL, SMX, and SQX)	60°C, 70°C, 80°C, 90°C and 100°C for 0, 30, 60, 90, 120, 150 and 180 min	LC–MS/MS	Stability during pasteurization (63°C for 30 min and 72°C for 15 s), and UHT (140°C for 4 s); Losses in the concentrations of sulfadimethoxine (6.5%) and sulfamethazine (85.1%) at 120°C for 20 min	Roca et al. (2013)
12	100 µg/L (OTC and TYL)	(i) 63°C for 30 min and (ii) 72°C for 15 s	LC–MS	TYL with 32% degradation by HTST; acidification and heating of whey decreased OTC concentration (14.7%–46.3%), and increased TYL concentration (184%–197%)	Hassan et al. (2021)
13	50, 100, 200 ng/mL (PEN G)	72°C for 15 s (HTST), 62.5°C for 30 min (LTLT) and 138°C for 2 s (UHT)	HPLC‐UV	Reduction of PEN‐G 13.5%, 6.1%, and 1.2% by HTST, LTLT, and UHT, respectively	Shinde et al. (2021)
17	25 ng/mL (0.5·MRL), 50 ng/mL (1·MRL), and 25 ng/mL (5·MRL) (PEN‐G, CLO, AMP, CEP, CEX, STM, NEO, SDZ, STZ, TC, and TRIM)	(i) 100°C for 5 s, (ii) 100°C for 120 s and (iii) 100°C for 300 s	LC–MS/MS	Cefoperazone and cloxacillin proved to be the least and the most heat‐stable substances, with 78.3% and 9.6% degradation in 300 s; high heat stability when treated for a few seconds at around 100°C. Aminoglycosides exhibited intermediate (33.8%–43.6%), tetracycline (30.4%), and trimethoprim (22.6%) intermediate to high heat stability	László et al. (2018)
20	3, 6, and 12 µg/kg (PEN G); 4, 8, and 16 µg/kg (AMP, AMO); 60, 120, and 240 µg/kg (CLO); 55, 110, and 220 µg/kg (CEP); 100, 200, and 400 µg/kg (CEFQ); 65, 130, and 260 µg/kg (CEXM); 80, 160, and 220 µg/kg (CEX); 15, 30, and 60 µg/kg (CEL)	(i) 40°C for 10 min and 83°C for 10 min, (ii) 60°C for 30 min, (iii) 120°C for 20 min, and (iv) 140°C for 10 s	Bioassay (based on inhibition of *Geobacillus stearothermophilus* var. *calidolactis*)	Insignificant inactivation (40°C for 10 min); slight loss of antimicrobial activity (60°C for 30 min, and 140°C for 10 s); Inactivation of 20% of penicillin G, 27% of cephalexin, and 35% of cefuroxime (83°C for 10 min) and inactivation of penicillin (65%) and cephalosporins (90%) at 120°C for 20 min	Zorraquino et al. (2008)
22	50, 100, 200 µg/L (GEN); 300, 600, 1.200 µg/L (CAN), 200, 400, 800 µg/L (NEO); and 200, 400, 800 µg/L (STM)	(i) 60°C for 30 min, (ii) 120°C for 20 min, and (iii) 140°C for 10 s	Bioassay (based on the inhibition of *Bacillus subtilis* BGA)	Not inactivated at 60°C for 30 min, inactivation of 95% at all concentrations at 120°C for 20 min and inactivation of kanamycin (17%) and neomycin (40%) at 140°C for 10 s	Zorraquino et al. (2009)
24	150 µg/L (1.5* MRL) (TC and OTC)	85°C for 3 s	HPLC	Decrease by 5.74% and 15.3% in tetracycline and oxytetracycline residues, respectively	Kellnerová et al. (2014)
26	20, 40, and 80 µg/L (ERY); 100, 200, and 400 µg/L (SPI); 500, 1000, and 2000 µg/L (TLY) and 1000, 2000 and 4000 µg/L (LIN)	(i) 60°C for 30 min, (ii) 120°C for 20 min, and (iii) 140°C for 10 s	Bioassay (based on growth inhibition of *Micrococcus luteus*) Delvotest SP	Reduced activity of erythromycin (21%) and spiramycin (13%) at 60°C for 30 min; Inactivation of erythromycin (93%), spiramycin (64%), tylosin (51%) at 120°C for 20 min; inactivation of 30% (erythromycin), 35% (spiramycin), 12% (tylosin) at 140°C for 10 s). For lincomycin, inactivation of 5% lincomycin at 120°C for 20 min and 140°C for 10 s	Zorraquino et al. (2011)
27	50 and 100 µg/kg (OTC)	Thermal treatment was performed in batch using a circulating water jacket at 68°C and 12°C	LC‐HRMS	The thermal treatment performed did not reduce the concentration of OTC, both in half MRL and MRL spiked ovine milk samples	Cabizza et al. (2018)
28	500, 750, and 1000 ppm (PEN G, OTC, GEN, and sulfonamide)	(i) 60°C for 15 s, (ii) 65°C for 30 min, and (iii) 110°C for 10 min	Disc diffusion assay method (*B. subtilis*)	Oxytetracycline, gentamycin, and penicillin G residues in milk are reduced with pasteurization at 65°C and sterilization (at 110°C). Furthermore, sulfonamide is more thermally stable than oxytetracycline at 110°C	Khaskheli et al. (2021)
31	NI	(i) 63°C for 30 min, (ii) 100°C for 5 min, and (iii) 100°C for 5 min (microwave processing)	Disc diffusion assay method (*B. subtilis*)	Boiling and microwave heating were significantly more destructive than pasteurization to ARs in milk samples	Almashhadany (2021)
34	NI (OTC)	Boiling (5 min)	Spectrophotometric	Reduction of OTC level by boiling (8.32%–68.28%)	Faried et al. (2024)
36	0.09–1.07 IU/mL (PEN)	(i) 60°C for 30 min, (ii) 70°C for 30 min, (iii) 121°C for 15 min; and storage for 1, 3, and 7 days	Disk diffusion assay	Reduction of 0.9%–16.1% of penicillin in milk. The storage losses of the antibiotic were less in the milk samples heated at higher temperatures than in the raw samples or the samples pasteurized at 60°C	Shahani et al. (1995)
38	50, 100, 200 ng/mL (SMTZ)	(i) 62.5°C for 30 min (LTLT), (ii) 72°C for 15 s (HTST), and (iii) 138°C for 2 s (UHT)	HPLC	Reduction of sulfamethazine 7.3% by LTLT, 5.2% by HTST, and 4.6% by UHT	Shinde et al. (2022)
39	At and above the MRL (PEN, CLO, OXA, DIC, AMP, or NAF)	Heat treatment: 15 min at 90°C, fermentation (*Streptococcus salivarius* subsp. *thermophilus* and *Lactobacillus delbrueckii* subsp. *bulgaricus*) for 43°C for 8 h	LC‐UV; LC–MS/MS, ELISA, and Charm II test	Reduction of penicillin levels	Grunwald and Petz (2003)
40	NI (OTC and SMTZ)	Boiling milk (100.17°C) for 2 or 5 min	Diffusion Assay (*B. subtilis*); high‐performance liquid chromatography–ultraviolet detector (HPLC–UV)	30.5% and 54.1% reduction of OTC in milk boiled for 2 and 5 min, respectively, 1.7% and 9.5% reductions of SMZ in milk boiled for 2 and 5 min, respectively	Fathy et al. (2019)
41	200 ppb (OTC and TC) and 400 ppb (OTC)	63°C for 30 min	HPLC with UV detector	Reduction in OTC (9.36%–86.17%), TC (22.97%–54.15%), and CTC residues in milk	Loksuwan (2002)
42	4 µg/kg (PEN G)	Heat treatment: 75°C, 85°C, 90°C, 92°C, and 95°C for 10, 30, 60, 90, and 120 min	Delvotest BR Brilliant	Only heating to 95°C for 120 min led to a degradation of 4 µg/kg penicillin G	Nitz et al. (2025)
43	200 µg/L (ENR)	(i)72°C for 15 s (HTST), (ii) 89°C for 1 s (HHST), (iii) 121°C for 15 min	HPLC	Highest percentage of antibiotic residue degradation is heating at 121°C for 15 min (6.34%)	Widiyanti et al. (2022)
44	NI (PEN)	90°C for 52 min	Delvotest T	Degradation of Penicillin levels.	Sats et al. (2014)
45	3, 6 and 9 ppm of TYL, ENR, AMP, and OTC each	(i) 63°C for 30 min, (ii) boiling: 87°C for 5 and 10 min	LC–MS/MS	TYL was affected by pasteurization (49.3%–15%), than OTC (46%–35.3%), ENR (44.6%–32%), and AMP (30.6%–26.6%). The pasteurization treatment was more effective in reducing antibiotic concentration than the boiling treatment. In addition, long‐time boiling (10 min) is better than short (5 min)	Alawad et al. (2025)
46	40 mg/kg of TC and STZ	72°C for 30 min	HPLC and LC–MS	Reductions (47.5%) for TC, no reduction was observed for STZ	Sidirokastritis et al. (2022)
49	1.99 mg/kg (SMZ)	(i) Pasteurization: 65°C for 30, 45, and 60 min and 72°C for 15 s, 2 and 10 min; (ii) boiling: 100°C for 2, 5, and 10 min; (iii) sterilization: at 121°C for 10, 15, and 20 min	LC	Range reductions of SMZ: Boiling (8.91%–18.56%) and sterilization (18.56%–21.53%). No reduction of SMZ concentration in incurred milk samples was observed during pasteurization	Papapanagiotou et al. (2005)

Abbreviations: AMO, amoxicillin; AMP, ampicillin; CAN, canamicin; CEF, ceftiofur; CEFQ, cefquinome; CEL, cephalonium; CEP, cefoperazone; CEPI, cephapirin sodium salt; CEX, cephalexin; CEXM, cephuroxime; CIP, ciprofloxacin; CLO, cloxacillin; CTC, chlortetracycline; DAN, danofloxacin; DC, doxycycline; DEM, demeclocycline; DIC, dicloxacillin; DIF, difloxacin; ENR, enrofloxacin; ERY, erythromycin; FLU, flumequine; GEN, gentamicin; HHST, high‐heat shorter time pasteurization; HTST, high‐temperature short‐time pasteurization; LIN, lincomycin; LTLT, low‐temperature long‐time; MAR, marbofloxacin; METC, methacycline; MINO, minocycline; NAF, nafcillin; NEO, neomycin; NOR, norfloxacin; OTC, oxytetracycline; OXA, oxolinic acid; PEN G, penicillin G; SAR, sarafloxacin; SCL, sulfachloropyridazine; SDZ, sulfadiazine; SMM, sulfamonomethoxine sodium; SMR, sulfamerazine; SMTZ, sulfamethazine; SMX, sulfadimethoxine; SPD, sulfapyridine; SQX, sulfaquinoxaline; STM, streptomycin; STZ, sulfathiazole; TC, tetracycline; TRIM, trimethoprim; TYL, tylosin.

The presented results reveal an uneven distribution in the occurrence of different strategies investigated for the mitigation of ARs. The notable predominance of thermal methods may be attributed to their established nature and widespread implementation in milk and dairy processing industries.

**FIGURE 12:**
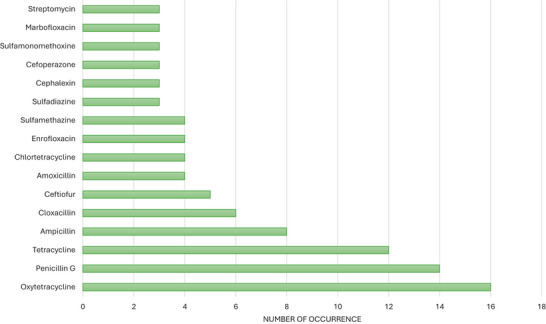
Frequency of antibiotic residues in the included studies.

Regarding the antibiotics studied in the included articles, the β‐lactam class (penicillin and cephalosporins) and tetracyclines were the most investigated (Figure 12). OTC was the most common, followed by penicillin G, tetracycline, ampicillin, chlortetracycline, cloxacillin, and amoxicillin. This result is expected, given that these antibiotics have been widely used in the treatment of dairy cattle (Priyanka et al. 2017). Studies on the occurrence of ARs in milk have shown a higher prevalence of penicillin G, amoxicillin, tetracycline, and OTC (Gwandu et al. 2018; Treiber and Beranek‐Knauer 2021; Simbine‐Ribisse et al. 2024). However, this high concentration of the tetracycline and β‐lactam classes may lead to a gap in knowledge about the mitigation of other relevant antibiotic classes, such as quinolones, macrolides, and sulfonamides, which have also been addressed, but less frequently.

### 3.4 Comparative and Critical Analysis of Mitigation of ARs in Dairy Products

#### 3.4.1 Thermal Processing Technologies on Mitigation of ARs in Dairy Products

Historically, the primary objective of heat treatment in milk is to ensure microbiological safety due to its ability to reduce microbial growth (Neoκleous et al. 2022; J. Chen et al. 2024; Omairi et al. 2022). However, the efficacy of these methods for ARs mitigation is highly variable as it depends on the specific antibiotic's thermostability, as well as the treatment temperature and duration. According to Roca et al. (2013), antibiotic degradation by high temperatures is a chemical process governed by the principles of chemical kinetics. Heat increases the kinetic energy of molecules, which elevates the frequency and energy of intermolecular collisions. When the energy of a collision exceeds the activation energy required for a specific reaction, such as bond cleavage, the antibiotic molecule can be degraded. This process can occur through various chemical pathways, including hydrolysis, oxidation, epimerization, decarboxylation, and dehydration, leading to antibiotic inactivation or transformation into other compounds.

A comparative analysis of these methods reveals significant trade‐offs, which are summarized in Table 4. The conditions optimized to destroy pathogens are, in most cases, insufficient to chemically degrade ARs, especially the thermostable ones. Standard pasteurization techniques, like low‐temperature long‐time (LTLT, 60°C for 30 min) and high‐temperature short‐time (HTST), show limited to moderate success, often achieving less than a 30% reduction for thermostable compounds such as tetracyclines and sulfonamides. In contrast, more intense treatments, like UHT (140°C for 10 s) and sterilization (120°C for 20 min), can achieve much higher degradation rates, ranging from 30% to over 90% (Roca et al. 2011; Zorraquino et al. 2009, 2008, 2011). Despite their effectiveness, these high‐intensity treatments come at a significant cost, causing damage to the milk's nutritional and sensory profile, including vitamin loss and Maillard reactions, making it commercially unviable. Furthermore, it is critical to note that simple boiling, a common household practice, is largely ineffective for many residues and can even concentrate them, representing a point of public health misinformation that warrants clarification. The heavy research focus on thermal methods reflects their incumbent status in the industry, yet their limitations highlight the clear need for effective alternatives.

**TABLE 4:** Comparative summary of thermal mitigation methods for ARs in milk. The table presents the efficacy and limitations of different thermal processing methods, including pasteurization (HTST and LTLT), UHT treatment, sterilization, boiling, and microwave heating. Efficacy is expressed as a percentage reduction of antibiotic residues under specified conditions.

Method category	Specific method	Antibiotic class	Applied conditions	Efficacy (% reduction)	Limitations
Pasteurization	HTST	Penicillins, cephalosporins, tetracyclines, sulfonamides, macrolides	72°C for 15 s	Variable: <10% for sulfonamides; >70% for penicillins	Ineffective for thermostable antibiotics (e.g., tetracyclines, sulfonamides). Its efficacy is highly dependent on the chemical structure of the evaluated antibiotic
	LTLT	Penicillins, cephalosporins, tetracyclines, sulfonamides, macrolides	63°C–65°C for 30 min	Variable: Penicillins (6.1%–16.1%), cephalosporins (27%–100%), tetracyclines (6.0%–86.2%), sulfonamides (<10%), macrolides (13%–49.3%)	Even less effective than HTST due to lower temperatures. Practically useless for the majority of antibiotics
UHT treatment	UHT	Sulfonamides	140°C for 10 s	Moderate to high: 83.1% for sulfamethoxazole (SMX), but only 6.5% for sulfamethazine (SMZ). Stability varies greatly within the same antibiotic class	More effective than pasteurization, but still insufficient for complete degradation
Sterilization	Sterilization	Cephalosporins, penicillins, tetracyclines, sulfonamides	120°C for 20 min	High: Up to 100% degradation for cefoperazone; 90% for ampicillin	Most effective method, but industrially unfeasible for fluid milk due to drastic alterations in sensory and nutritional characteristics. Causes degradation of vitamins and proteins
Boiling	Boiling	Quinolones, tetracyclines, sulfonamides	100°C for 5–10 min	Moderate: Quinolone (∼12.7%); sulfonamide (30%–54%), tetracycline (8.32%–68.3%). Prolonged boiling time increases degradation	More effective than pasteurization but can negatively impact the nutritional and sensory properties of milk
Other	Microwaves	Penicillins, sulfonamides	100°C for 5 min	Destructive, more effective than pasteurization	Causes significant alterations in milk. Difficulty in industrial‐scale application and ensuring uniform heating

Abbreviations: HTST, high‐temperature short‐time; LTLT, low‐temperature long‐time; UHT, ultrahigh temperature.

#### 3.4.2 Cold Storage on Mitigation of ARs in Dairy Products

Refrigeration (cold storage) and freezing (below −18°C) are preservation methods primarily intended to slow microbial and biochemical degradation, such as lipid oxidation (M. Chen et al. 2016). However, some studies have demonstrated that these processes can induce the passive degradation of certain ARs. This degradation is not an active, controlled process but rather a consequence of the inherent instability of some molecules over time at low temperatures.

The efficacy of this passive degradation is highly variable and depends on the antibiotic class, as summarized in Table 5. For instance, OTC showed a promising reduction of approximately 80% (Faried et al. 2024). In contrast, quinolones proved more stable, with reductions of up to 30% (M. Chen et al. 2016), while sulfonamides demonstrated high stability during frozen storage, with no significant reductions occurring (Papapanagiotou et al. 2005). Although many antibiotics are stable in frozen food samples (Różańska and Osek 2013), the application of these methods as an industrial‐scale mitigation strategy is unfeasible for three main reasons: (1) the prolonged time required to achieve the desired degradation; (2) the high energy costs associated with long‐term storage; and (3) the potential negative impact on milk quality during freezing, which would reduce the product's commercial value

**TABLE 5:** Summary of key studies investigating the stability of antibiotic residues in milk under various storage conditions.

Study no.	Concentration on target antibiotics	Conditions	Method for identification	Main results	References
3	15, 30, and 100 µg/kg (CLO)	Storage: (i) 4°C–8°C for 7 days and (ii) 20°C for 30 days	UHPLC/HPLC	Cloxacillin residues were stable in yogurt, and residue reduction was in the range of 5%–10%, with a relative response ≥ 90%	Gbylik‐Sikorska et al. (2021)
8	100 µg/L (CIP, ENR), 30 µg/L (DAN), 50 µg/L (FLU, DIF, SAR)	(i) 4°C for 4, 8, 24, and 48 h; (ii) −20°C and −80°C for 1, 7, and 30 days; (iii) thawed at 25°C, 40°C, and 60°C after storage at −20°C for 24 h; (iv) freeze–thawed for one, three, and five times after storage at −20°C with a thawing temperature of 40°C	UPLC–MS/MS	Degradation of quinolones in raw milk increased the longer the samples were stored at 4°C; no degradation of quinolones was seen when milk samples were stored at −20°C for up to 7 days; storage at −20°C and −80°C resulted in about 30% of degradation; no losses were observed when frozen milk samples were thawed at 25°C, 40°C, or 60°C	Chen et al. (2016)
30	1× MRL, 1.5× MRL, and 2× MRL (100 ppb) for PEN G, AMO, CLO, and CEF; 100 ppb (MRL), 500 ppb, and 700 ppb for OTC	Storage at 4 ± 2°C and −18 ± 2°C	Delvotest SP‐NT and receptor assay CHARM ROSA MRL BL/TET	The lowest durability was observed for penicillin G and oxytetracycline. In cooled samples, antibiotics were detected up to 72 h. The penicillin G concentration reduces to 4 ppb after 7days in frozen samples	Rózańska and Osek (2013)
34	NI (OTC)	Freezing (−20°C for 24 h)	Spectrophotometric	Reduction of OTC level by freezing (2.58%–81.63%)	Faried et al. (2024)
49	1.99 mg/kg (SMZ)	(i) −20°C at 3 months and (ii) −75°C at 5 months	LC	SMZ concentration remained unchanged after storage	Papapanagiotou et al. (2005)

Abbreviations: AMO, amoxicillin; CEF, ceftiofur; CIP, ciprofloxacin; CLO, cloxacillin; DAN, danofloxacin; DIF, difloxacin; ENR, enrofloxacin; FLU, flumequine; MRL, maximum residue limits; NI, not indicate; OTC, oxytetracycline; PEN G, penicillin G; SAR, sarafloxacin.

#### 3.4.3 Nonthermal Emerging Processing Technologies on Mitigation of ARs in Dairy Products

As described in Section 3.4.1, treatment is a classic method widely used by industries, including the dairy industry; however, despite its positive effects, this technique is having a depletory impact related to the alteration of milk's chemical composition and, consequently, sensory changes in flavor, aroma, and nutritional value (Pegu and Arya 2023; Neoκleous et al. 2022). Given these limitations associated with ensuring food safety, the food industry is seeking alternative methods to conventional treatment (Sersa and Cemazar 2023; J. Chen et al. 2024). Thus, recently, much research has been directed toward this topic.

The use of emerging nonthermal technologies such as PEF, high pressure processing (HPP), supercritical carbon dioxide (SC‐CO_2_), UV light, CP, ozone treatment (O_3_), ultrasound technology, and PL has been the subject of much research (Lopes et al. 2023; Misra 2015), applied as an interesting and promising alternative for food processing, including liquid products (Redondo 2024; Jiang 2024; Zare et al. 2023; Lopes et al. 2023; Shinde et al. 2021; Feng et al. 2022) such as milk (Pegu and Arya 2023; Neoκleous et al. 2022; J. Chen et al. 2024).

Literature review data demonstrate that studies on emerging nonthermal methods applied to milk have primarily documented two fundamental aspects: the antimicrobial potential of these techniques and their effects on preserving milk quality. Technologies such as HPP, PEF, UV, CP, IOR, PL, O_3_, and ultrasound are widely used in the dairy industry, with significant reductions in microbial load reported in the raw material (Pegu and Arya 2023; Neoκleous et al. 2022). Other studies have systematically evaluated the impact of these technologies on the chemical composition of milk, confirming their ability to maintain the organoleptic characteristics (flavor, aroma, texture, and appearance) and nutritional properties of the product (J. Chen et al. 2024).

Nowadays, research has expanded the scope of application of nonthermal technologies beyond microbiological safety, investigating their potential in the decontamination of undesirable chemical substances present in food, such as pesticides (Misra 2015) and mycotoxins (Gavahian et al. 2021; Nunes et al. 2021). The application of these technologies in the mitigation of ARs in milk has already been proven with consistent results, where treatments such as PEF (Shinde et al. 2021, 2022), O_3_ (Alsager et al. 2018; Sert and Mercan 2021), and sonication (Liu et al. 2023) have demonstrated the ability to reduce concentrations of antibiotics such as sulfonamides, tetracyclines, and β‐lactams by up to 90% (Table 6).

**TABLE 6:** Emerging nonthermal processing technologies in antibiotic residues mitigation in milk. The Table presents a summary of experimental conditions, target antibiotics, detection technique, main findings, and references.

Methods	Experimental conditions	Antibiotic (concentration)	Detection technique	Main results	Reference
Sonication	Frequency: 500 kHz Power: 259 W Time: 130–150 min	CEF, SMM, MAR, and OTC (192.8 µM)	HPLC; Kirby–Bauer disk diffusion	After 600 min of sonication, the observed degradation percentages were 16.9% for CEF, 12.2% for SMM, 11.4% for MAR, and 10.9% for OTC. Further experiments focusing on SMM (initial concentration 6.62 µM) in a milk matrix showed that sonication for 130–150 min reduced its residual concentration to the range of 52.9–96.3 µg/L. Notably, the addition of 0.5 mM hydrogen peroxide (H_2_O_2_) was found to accelerate the degradation of SMM in milk by 33%	Liu et al. (2023)
	Frequency: 500 kHz	CEF, SMM, MAR, and OTC (5.52 µmol/L)	HPLC	Synergism between adsorption and sonication during sonocatalysis	Liu et al. (2024)
	Power: 259 W				
	Time: 20–60 min				
	Addition of 20 mg activated carbon				
	Room temperature				
Ozonation	Flow rate: 0.75 L/min	AMO, DC, CIP, and SDZ (200 µM/75 mg/L)	HPLC UV–VIS	The efficiency of antibiotic removal demonstrated a positive correlation with the applied ozone concentration, showing an increase as the dose was raised from 15 to 37.5 mg/L. Furthermore, a high removal rate of 95% was achieved for the target antibiotics when an ozone dose of 75 mg/L was applied	Alsager et al. (2018)
	Time: 0, 2, 5, and 10 min				
	Room temperature 23°C				
	Time: 5, 10, 15, 30, or 60 min; capacity of 3.5 g ozone/h	β‐Lactam and tetracycline	BetaStar S Combo	After 60 min of ozone treatment, both β‐lactam and tetracycline (TC) residues in the milk samples were below the limit of detection	Sert and Mercan (2021)
	Temperature: 20 ± 2°C				
	Ozonation in the vortex reactor	CEF, SMM, MAR, and OTC (5.52 µM)	HPLC	ABX removal via ozonation is better using stronger vortexing, which induces hydrodynamic cavitation. CEF undergoes the fastest degradation, followed by SMM, MAR, and OTC. After the ozonation of 400 mL of 5.52 µM various ABX for 20–40 min, their residue concentrations (50.8–84.1 µg/L) satisfy the relevant MRLs	Liu et al. (2022)
	Water flow (*Q*, L/h): 467–1980				
	Flow velocity in the tube (*v*, m/s): 0.37–1.59				
	Circulation frequency (h^−1^): 584–2476				
	50 kHz, 200 W, 25°C	TC and SMTZ (40 mg/kg)	HPLC	Reduction of 4.1 for SMTZ and 29.8 for TC	Sidirokastritis et al. (2022)
Pulsed electric field (PEF)	Voltage: 20%–65%	PEN G (50, 100, 200 ng/mL)	HPLC	Reduction of PEN G 13.5%, 6.1%, and 1.2% by HTST, LTLT, and UHT, respectively. PEF‐induced reduction efficiency achieved was 79%–86%. The reduction percentages were observed to be higher in the combined pasteurization and PEF‐treated milk (86%–91%)	Shinde et al. (2021)
	Pulse: 10–26 µs				
	Maximum frequency: 1 kHz				
	Flow rate: 41.5 L/h.				
	Combined: 72°C for 15 s (HTST), 62.5°C for 30 min (LTLT), and 138°C for 2 s (UHT)				
	Voltage: 20%–65%;	SMTZ (50, 100, 200 ng/mL)	HPLC	Reduction of sulfamethazine 7.3% by LTLT, 5.2% by HTST, and 4.6% by UHT. PEF and a combination of thermal + PEF reduce sulfamethazine by 67%–72% and 73%–76%, respectively	Shinde et al. (2022)
	Pulse width: 10–26 µs				
	Maximum frequency: 1 kHz				
	Flow rate: 41.5 L/h				
	Frequency: 25 kV/cm, pulse width: 172.8–345.6 µs	OTC (50, 100, and 200 µg/kg)	HPLC–MS/MS	Degradation rate of 85.01%	Wen et al. (2025)
High hydrostatic pressure (HHP)	Pressure: 150–580 MPa, 6 min, vacuum condition (50 mbar)	TC and SMTZ (40 mg/kg)	HPLC	Reduction of 64.8%–84.3% for SMTZ and 57.7%–70.3% for TC	Sidirokastritis et al. (2022)

*Ozonation* represents one of the most promising methods for the removal of ARs in milk, as the process degrades antibiotics into nontoxic products, eliminating their residual antimicrobial activity (Varga and Szigeti 2016; Liu et al. 2022). Recent studies have demonstrated the high efficiency of applying ozonation for the removal of ARs from milk (Alsager et al. 2018). According to Silva et al. (2022), ozone (O_3_) is a powerful electrophilic oxidant, surpassed only by the hydroxyl radical (•OH), which reacts rapidly with organic molecules containing nucleophilic groups, such as carbon–carbon double bonds, aromatic rings, and functional groups containing sulfur, phosphorus, nitrogen, and oxygen. For Iakovides et al. (2019), this characteristic is unique and makes ozone efficient for the degradation of antibiotics, which often have multiple reactive sites in their molecular structures. The ozonation process can occur directly through molecular ozone or indirectly through •OH radicals formed in situ in aqueous matrices. Liu et al. (2022) investigated the use of ozonation in a vortex reactor to remove ceftiofur hydrochloride (CEF), sulfamonomethoxine sodium (SMM), marbofloxacin (MAR), and OTC from milk. The results obtained by these authors showed that ozonation is more efficient with a stronger vortex, which induces hydrodynamic cavitation. The reported order of antibiotic degradation was CEF > SMM > MAR > OTC. The authors suggest that the high hydrophobicity of antibiotics favors degradation via ozonation. Alsager et al. (2018) reported the removal of antibiotics (amoxicillin, doxycycline, ciprofloxacin, and sulfadiazine) at around 95% for all tested compounds with an ozone dose of only 75 mg/L. These authors observed that the removal of antibiotics in milk samples was more efficient, with faster rate constants compared to aqueous solutions. This phenomenon was attributed to the self‐buffering characteristic of milk, which maintains a neutral pH, keeping the amine groups unprotonated and more reactive to electrophilic attack by molecular ozone. Despite its efficacy in reducing ARs, the application of ozonation in milk presents significant challenges. The literature emphasizes that, in dairy systems, the primary concerns are related to oxidative effects on the milk matrix, particularly lipid and protein oxidation, which may lead to adverse sensory changes at high ozone concentrations (Vashisht et al. 2024; Botondi et al. 2023). While the formation of toxic by‐products such as bromate is a well‐documented concern in water treatment, arising from the oxidation of bromide ions (Gounden and Jonnalagadda 2019; Morrison et al. 2023)‐its occurrence and toxicological relevance in milk have not been systematically demonstrated, consistent with milk not being identified as a bromide‐rich matrix. This distinction underscores a critical principle: reaction pathways from simple aqueous systems cannot be directly extrapolated to complex food matrices. Therefore, from a precautionary standpoint, the main focus remains on the precise control of ozone dose and exposure time to prevent excessive oxidation of milk components (Botondi et al. 2023). Regulatory approval also remains a major hurdle in many regions. While the United States by Food and Drug Administration (FDA) has designated ozone as Generally Recognized as Safe (GRAS) for all food products, including milk, since 2001 (FDA 2002) specific dosage guidelines are lacking, requiring adherence to good manufacturing practices. The European Union regulates ozone as a disinfectant, and for workplace safety, OSHA has set an exposure limit of 0.1 ppm for 8 h (Botondi et al. 2023, Vashisht et al. 2024).

*PEF* is an emerging nonthermal technology that has shown promising capabilities for the degradation of complex organic compounds, including ARs. This technology is based on the application of high‐intensity electric pulses (10–80 kV/cm) for extremely short periods (microseconds to milliseconds), inducing electrochemical and physicochemical phenomena that result in the degradation of organic molecules without the need for significant heating (Misra 2015). Studies have proven that the application of PEF results in fresher products with maintained flavor and color properties, as well as high nutritional value (Redondo 2024; Zare et al. 2023). The efficiency of using PEF in reducing pesticide residues in fruit juices (F. Chen et al. 2009; Misra 2015; Zhang et al. 2012) and ARs in milk (Shinde et al. 2021, 2022) has already been proven. Shinde et al. (2022) conducted a comprehensive study on the effect of PEF processing on the reduction of sulfamethazine in whole milk. These authors reported that the PEF treatment of milk showed significantly higher efficiency, achieving reductions of 67%–72% of the sulfamethazine content. Evaluating the effect of PEF application on benzylpenicillin in milk, Shinde et al. (2021) reported an efficiency in reducing this antibiotic in the range of 79%–86%, while maintaining the quality of the milk. PEF is a promising technology for the dairy industry, but its large‐scale application faces significant technical, economic, and regulatory challenges (Lyng et al. 2022). While effective in laboratory studies, the transition to industrial scale is complex due to issues of treatment uniformity, heat conduction, and residence times. Processing costs are likely higher for dairy products due to their high electrical conductivity, requiring premium‐priced products to justify the investment. Key obstacles to commercialization include the need for robust equipment (generators and electrodes), high investment and maintenance costs, and consumer acceptance (Cavalcanti et al. 2023). The technology appears most viable for niche products such as infant formulas, colostrum, and bioactive dairy beverages, where preservation of heat‐sensitive compounds adds value. Furthermore, the potential formation of undesirable by‐products from the interaction of the electric field with the milk matrix is an area that warrants further investigation (Buckow et al. 2014; Cavalcanti et al. 2023).

From a regulatory perspective, the landscape is still evolving. In the United States, the FDA permits PEF use for juices, requiring a 5‐log reduction of the most resistant pathogen, a standard that would also be expected for milk (Lyng et al. 2022). However, specific regulations for milk pasteurization using PEF are not yet universally established (Cavalcanti et al. 2023). In the European Union, the regulatory approach focuses on demonstrating equivalence with traditional methods, such as HTST pasteurization (Buckow et al. 2014).

*Sonication* is a widely researched technique for the degradation of ARs. Recent studies have demonstrated the effectiveness of this method in degrading various classes of antibiotics in milk, including cephalosporins, sulfonamides, fluoroquinolones, and tetracyclines. Liu et al. (2024) investigated the sonocatalytic degradation of four antibiotics in bovine milk and observed a significant synergistic efficiency when combining ultrasound with activated carbon. The study also confirmed that the degradation follows pseudo‐first‐order kinetics and that hydrophobic antibiotics are degraded more quickly. Another study by Liu et al. (2023) focused on the kinetics and mechanisms of sonolytic degradation of antibiotics in water and milk, revealing that degradation is significantly faster in water than in milk, due to matrix effects. However, even in milk, sonication proved to be effective, and no significant changes in milk nutrients were observed after the treatment. Sonication treatment demonstrated faster degradation of ceftiofur than sulfamonomethoxine, MAR, and OTC (Liu et al. 2023). While effective in laboratory settings, its industrial scalability is a major challenge. The process can be energy‐intensive, and the generation of localized high temperatures during cavitation can cause some of the same nutritional damage observed with thermal treatments. Its efficacy is also highly dependent on the frequency, power, and treatment time (Chávez‐Martínez et al. 2020).

The method's efficiency in degrading varies by specific antibiotics. However, these investigations are still incipient, requiring more comprehensive studies on mechanisms of action, parameter optimization, and evaluation of possible degradation by‐products.

#### 3.4.4 Biological Approach

Biological methods represent a promising frontier for mitigating ARs in milk, offering high specificity and the potential for integration into existing dairy processing workflows (Grunwald and Petz 2003). These more sustainable and natural approaches have been gaining significant attention for reducing chemical contaminants in milk (Gavahian et al. 2021; Nunes et al. 2021) and antibiotics (Grunwald and Petz 2003). These approaches primarily encompass two distinct but related strategies: direct enzymatic treatment using isolated enzymes and fermentation‐based degradation driven by complex microbial ecosystems (Mayo et al. 2021). This section provides a detailed examination of the mechanisms, microbial interactions, and practical challenges associated with these biological strategies, unifying fermentation and enzymatic treatment under a single comprehensive framework.

- Enzymatic treatment

The use of enzymes has also been evaluated for the mitigation of ARs in milk. Direct enzymatic treatment involves the application of specific, purified enzymes to inactive antibiotic molecules. This method is celebrated for its high specificity, where a particular enzyme is chosen to target a specific class of antibiotics, minimizing off‐target effects on milk components (L. Li et al. 2014). The most well‐documented example is the use of β‐lactamases applied for the degradation of penicillin and other β‐lactams antibiotics, through highly selective biochemical reactions. The mechanism of action is precise and well‐understood. β‐Lactamase catalyzes the hydrolysis of the amide bond within the characteristic four‐membered β‐lactam ring, rendering the antibiotic biologically inactive. This process yields primary degradation products such as penicilloic acid, which is unstable and can further degrade into more stable compounds like penilloic acid through decarboxylation (Horton et al. 2015; L. Li et al. 2014). L. Li et al. (2014) highlight that the use of enzymes in the mitigation of ARs in milk may be limited due to the high cost of acquisition, the specificity for each antibiotic, and the specific conditions required for optimal enzymatic activity (pH, temperature, ionic strength), which may not be compatible with the natural conditions of milk. While highly effective for its target, the primary limitation of direct enzymatic treatment is its narrow spectrum of activity. A specific enzyme like β‐lactamase is ineffective against other major antibiotic classes, such as tetracyclines or sulfonamides. Therefore, addressing a broader range of potential contaminants would require a cocktail of different enzymes, which introduces significant challenges related to cost, enzyme stability, and the optimization of reaction conditions for multiple enzymatic systems simultaneously.

ii. Fermentation

Fermentation, a cornerstone of dairy processing for products such as cheese (Quintanilla et al. 2020; Quintanilla et al. 2021) and yogurt (Grunwald and Petz 2003), also serves as a potent, albeit more complex, method for reducing ARs. Recently, biological mitigation methods based on microbial cells (bacteria or yeasts) have been widely recognized as effective and are increasingly garnering attention for reducing chemical compounds, such as mycotoxins, via adsorption or degradation (Gavahian et al. 2021; Nunes et al. 2021; Zhu et al. 2016). LAB are the most frequently studied microorganisms; numerous studies have demonstrated their positive effect in reducing mycotoxins (Gavahian et al. 2021; Mayo et al. 2021). However, studies on the role of fermentation in reducing ARs are still scarce (Adetunji 2011; Gbylik‐Sikorska et al. 2021; Grunwald and Petz 2003; Horton et al. 2015; Umam et al. 2023), and the mechanisms behind residue reduction remain poorly understood. It is known that the mechanisms are complex and that this process relies primarily on the metabolic activity of LAB, which modifies the milk matrix (pH change), associated with a combination of enzymatic degradation and physical binding of residues to bacterial cell walls. These conditions can alter antibiotic stability. Studies have shown that certain strains of Lactobacillus and Bifidobacterium can significantly reduce levels of tetracyclines and β‐lactams in fermented products like yogurt (Gbylik‐Sikorska et al. 2021; Mayo et al. 2021).

Grunwald and Petz (2003) showed that fermentation specifically for yogurt production leads to a reduction of ARs present in milk, such as penicillin. It should be noted, however, that various factors interact to enable this reduction during milk processing, including heat treatment before culture inoculation, fermentation temperature, fermentation duration, and the binding of antibiotics to milk proteins. Similar findings were reported by Adetunji (2011), who evaluated the effect of milk processing on residues of streptomycin, penicillin‐G, and tetracycline in cheese and yogurt production lines, showing that the transformation of milk into yogurt and cheese indeed reduced ARs levels—verified during fermentation—although the reduction was not statistically significant. Horton et al. (2015) demonstrated that cefquinome concentrations decreased significantly following fermentation at 37°C in the presence of probiotics or starter cultures (*Lactobacillus acidophilus* and *Enterococcus faecium*). Although it is a promising method, it is not without challenges. The efficacy is highly strain‐specific, meaning not all starter cultures are effective. The process is slower than chemical or physical methods, and the interactions between different microbial species in a complex culture can lead to unpredictable results (Mayo et al. 2021). Furthermore, this method is only applicable to fermented dairy products, not fluid milk, limiting its overall utility.

#### 3.4.5 Chemical Treatments

Chemical methods can be applied for the mitigation of ARs. In evaluating the included studies, it was noted that few authors have used chemical approaches for removing ARs in milk. Antibiotics belonging to the tetracycline (OTC, tetracycline, and chlortetracycline) and penicillin classes were the most investigated in the application of chemical methods. The use of hydrogen peroxide and electrochemical constitute the main methods evaluated in the included studies.

The use of hydrogen peroxide (H_2_O_2_) has demonstrated efficiency in removing ARs in aqueous solutions (Sarkale et al. 2024; Zhou et al. 2020; Nguyen et al. 2025). According to Hanway et al. (2005) the use of H_2_O_2_ in milk is an old technology, effective but highly controversial. Its use is strictly regulated and often restricted to emergencies (such as cooling system failure) due to significant concerns about its impact on product quality and safety, including high residual peroxide values. A concentration of 0.34% H_2_O_2_ was effective in removing penicillin G in milk to safe levels. Another proposed method is the electrochemical, which is based on the electrochemical oxidation of organic compounds by applying an electric current. The process involves oxidation reactions at the anode, where antibiotics are degraded through direct mechanisms resulting from oxidation on the electrode surface or indirect mechanisms through electrochemically generated oxidizing species (Kitazono et al. 2017). Inert electrodes (Ti/PbO_2_, Ti/RuO_2_–IrO_2_, or boron‐doped diamond “BDD”) are frequently employed due to their high stability and ability to generate potent oxidizing species (Kitazono et al. 2012; Kitazono et al. 2017). Kitazono et al. (2012) investigated the electrochemical oxidation of tetracycline antibiotics in bovine milk, focusing on the effects of anode materials and electrolytes on the degradation of OTC. The study demonstrated that a higher degradation rate of OTC was obtained using an inactive anode (Ti/PbO_2_) or NaCl electrolyte. Although this approach yields good results, its industrial use may be unfeasible and impractical due to its high cost and the need to add external agents such as acids and salts.

#### 3.4.6 Adsorption Methods and Novel Materials

Adsorption and selective capture offer a promising physical removal strategy, representing one of the most established and widely applied technologies for the removal of organic contaminants from aqueous solutions (Ma et al. 2022; Ajala et al. 2023). Our systematic review revealed that adsorptive methods, despite their potential, were among the least reported categories (5.1% of instances). However, this low frequency does not diminish the strategic importance of this approach but rather reflects the emerging state of advanced materials designed for this purpose.

The adsorption process is based on the transfer of antibiotic molecules from the liquid phase to a solid surface (adsorbent), where they are retained through physical and/or chemical interactions. Recent research has confirmed the positive effect of this technology on the removal of antibiotics in water (Stylianou et al. 2021; Ajala et al. 2023; Ma et al. 2022) and in milk (Jafari et al. 2019; Lu et al. 2021; Y. Li et al. 2025). The application of the adsorption technique for the removal of ARs in water is reported as a more common technique, related to its advantages such as low operational cost, and simple and practical operation (K. Li et al. 2020).

Activated carbon has unique characteristics that make it an effective adsorbent, including a high specific surface area, a well‐developed porous structure, resistance to acids and alkalis, and a strong and stable adsorption capacity. For this reason, it has stood out in the removal of ARs in aqueous solutions and milk, being the target of several research studies (Liu et al. 2024). Ge et al. (2021) demonstrated good efficiency of using commercial and modified activated carbons for the removal of MAR in water and milk. Liu et al. (2024) developed an analytical method based on solid‐phase extraction with activated carbon followed by HPLC for the determination of antibiotics in water and milk. The method demonstrated a recovery of over 99.0% for sulfamonomethoxine and OTC, highlighting the high affinity of these compounds for activated carbon.

The use of activated carbon presents many advantages, making this technology very promising and attractive, due to fundamental aspects such as the non‐production of toxic chemical by‐products (Ge et al. 2021). However, they suffer from a lack of selectivity, leading to the simultaneous removal of essential nutrients from milk (such as vitamins and fatty acids), thereby reducing its quality. This has driven research into new and more selective materials. Metal–organic frameworks (MOFs) and molecularly imprinted polymers (MIPs) are at the forefront of this research (Khezerlou et al. 2025; Niu et al. 2025). MOFs are crystalline materials with exceptionally high surface areas and tunable porosity. Their key advantage lies in the ability to design pore size and chemistry to selectively capture specific target molecules, such as a particular class of antibiotics. This intrinsic selectivity offers the potential to remove contaminants without affecting the complex nutritional matrix of milk (Khezerlou et al. 2025).

MIP operates on a “molecular memory” principle. They are synthetic polymers created around a template molecule (the antibiotic), forming recognition cavities with complementary shape and functionality. After the template is removed, the polymer retains tailor‐made “binding sites” that can selectively re‐bind the target antibiotic from a complex mixture, functioning analogously to a lock‐and‐key system (Tarannum et al. 2020).

Authors report that the efficiency of the adsorption process depends on several factors, including the properties of the adsorbent (surface area, pore distribution, surface functional groups), the characteristics of the adsorbate (molecular size, polarity, solubility), and the operational conditions (pH, temperature, contact time, concentration). Research has been developed to evaluate the efficiency of different adsorbent compounds for the removal of antibiotics, MIP (Jafari et al. 2019; Y. Li et al. 2025), MOF, such as structures based on (ZnCl_2_)_3_ (K. Li et al. 2020; Ma et al. 2022), and biomass (Stylianou et al. 2021; Suguna Devakumari 2021; Ajala et al. 2023). Niu et al. (2025) demonstrated the efficient removal (92%) of tetracyclines within 3 h using Fe‐MOF in milk. These materials can be designed with specific pore sizes and binding sites to selectively capture target antibiotic molecules. Despite their high potential, significant challenges remain. Their high production cost, the need to prove that they are food safe (i.e., no leaching of material components into milk), and the difficulty of regenerating and reusing them in a food‐grade industrial process currently limit their practical application.

**FIGURE 13:**
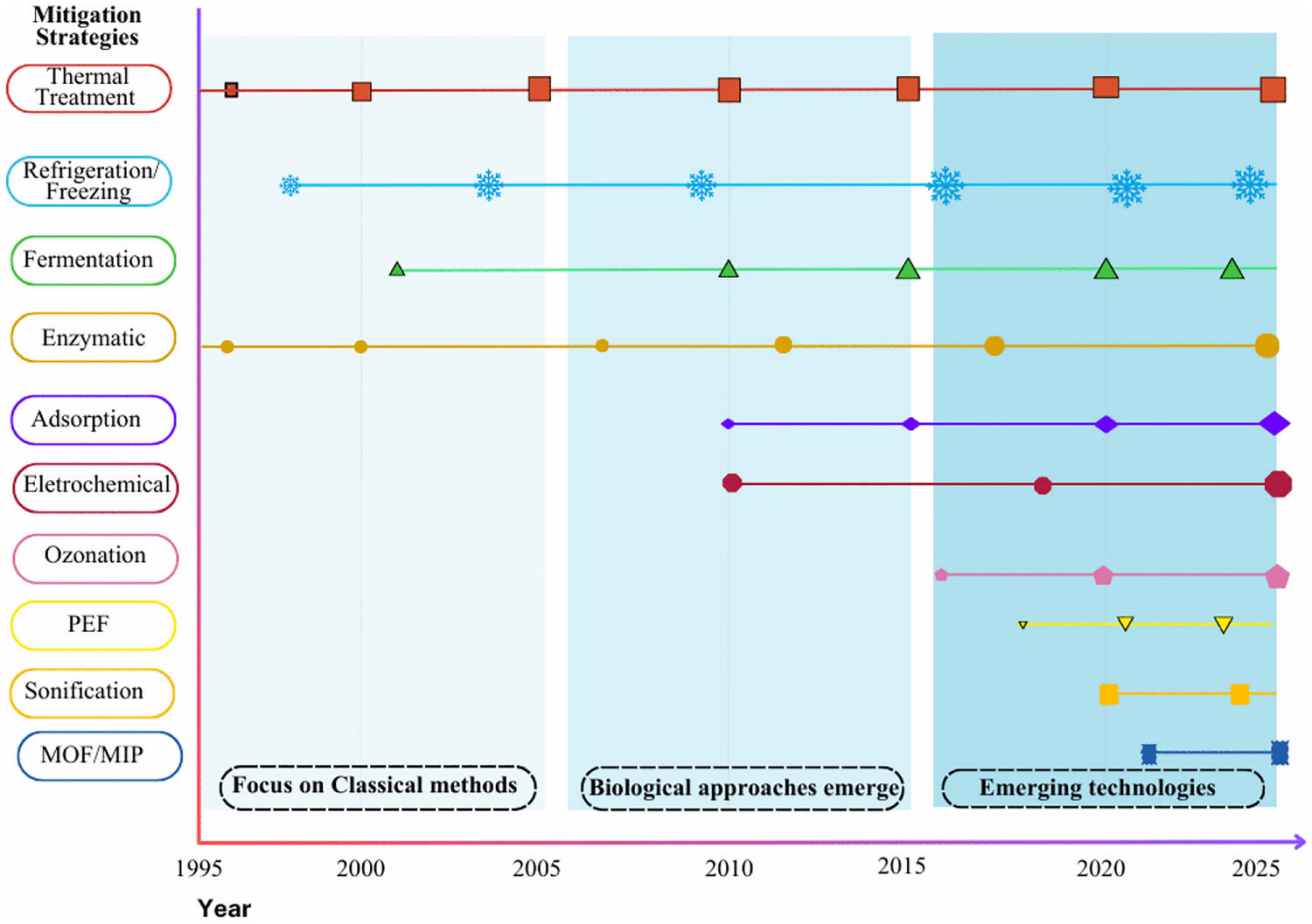
Temporal evolution of research focus on antibiotic mitigation strategies in milk (1995–2025). The timeline illustrates the emergence and development of different mitigation approaches over three distinct periods: Early period (1995–2005), characterized by conventional thermal methods; transition period (2006–2015), marked by the emergence of biological approaches; and recent period (2016–2025), featuring advanced and selective technologies. Bubble sizes represent relative research intensity (number of published studies), with larger bubbles indicating higher research activity. Color‐coded horizontal lines represent continuous research activity for each strategy, with different markers for easy identification. Visualization demonstrates a clear shift from predominantly thermal approaches to a diversified portfolio including biological, physical, and nanotechnology‐based methods.

The trajectory of research on ARs mitigation strategies in dairy products reveals significant progress over the last three decades, as summarized in Figure 13. The timeline shows a transition from an initial focus almost exclusively on thermal methods to the exploration of biological approaches and, more recently, to the development of advanced and selective technologies, such as adsorption nanotechnology (MOF/MIP) and nonthermal methods (PEF). This diversification indicates the maturation of the field of research and the search for increasingly effective and specific solutions.

### 3.5 Synergistic Effects of ARs Mitigation Strategies in Dairy Milk

The diversity of available strategies highlights that no single approach offers a universal solution for mitigating ARs in dairy products. An integrated approach combining multiple strategies in sequential or simultaneous systems represents the most promising avenue for comprehensive and effective mitigation of these residues, ensuring the safety and quality of dairy products. Several studies have pointed to the benefits of combining methods or adding intensifiers.

The addition of hydrogen peroxide (H_2_O_2_) significantly accelerated the degradation of Sulfamonomethoxine during sonication in milk by 33% (Liu et al. 2023). This suggests that intensifying hydroxyl radical (•OH) generation, likely through sonoFenton—type reactions facilitated by H_2_O_2_, is a viable strategy to increase sonolytic efficiency. Liu et al. (2024) explicitly noted a synergy between adsorption (using activated carbon) and sonication in sonocatalysis. The addition of commercial powdered activated carbon to the sonolytic system significantly improved the degradation efficiency of sulfamonomethoxine under specific conditions. This synergy likely arises because the adsorbent concentrates antibiotic molecules on its surface, bringing them closer to cavitation events and reactive species generated by ultrasound, thus increasing degradation rates.

The combination of PEF with thermal pasteurization also resulted in slightly higher removal efficiencies compared to PEF alone (Shinde et al. 2021, 2022). Although the increase was modest, it suggests that combined stresses may render antibiotics or matrix components more susceptible to degradation. Synergistic approaches—such as combining sonication with H_2_O_2_ or adsorption (sonocatalysis), and potentially PEF with pasteurization are also promising for increasing degradation efficiency.

Ge et al. (2021) improved the adsorption performance of a wood‐based powdered activated carbon (WPAC) through oxidation with H_2_O_2_ under different conditions: stirring, ultrasound, microwave heating, and microwave‐assisted calcination. The results showed that the ultrasound‐ and microwave‐assisted modifications led to a significantly superior adsorption performance for MAR compared to the activated carbon. The good adsorption performance was promoted by the presence of newly formed or converted oxygen‐containing functional groups during the modification of the activated carbon.

### 3.6 Relationship Between Main Antibiotics and Mitigation Strategies Applied

**FIGURE 14:**
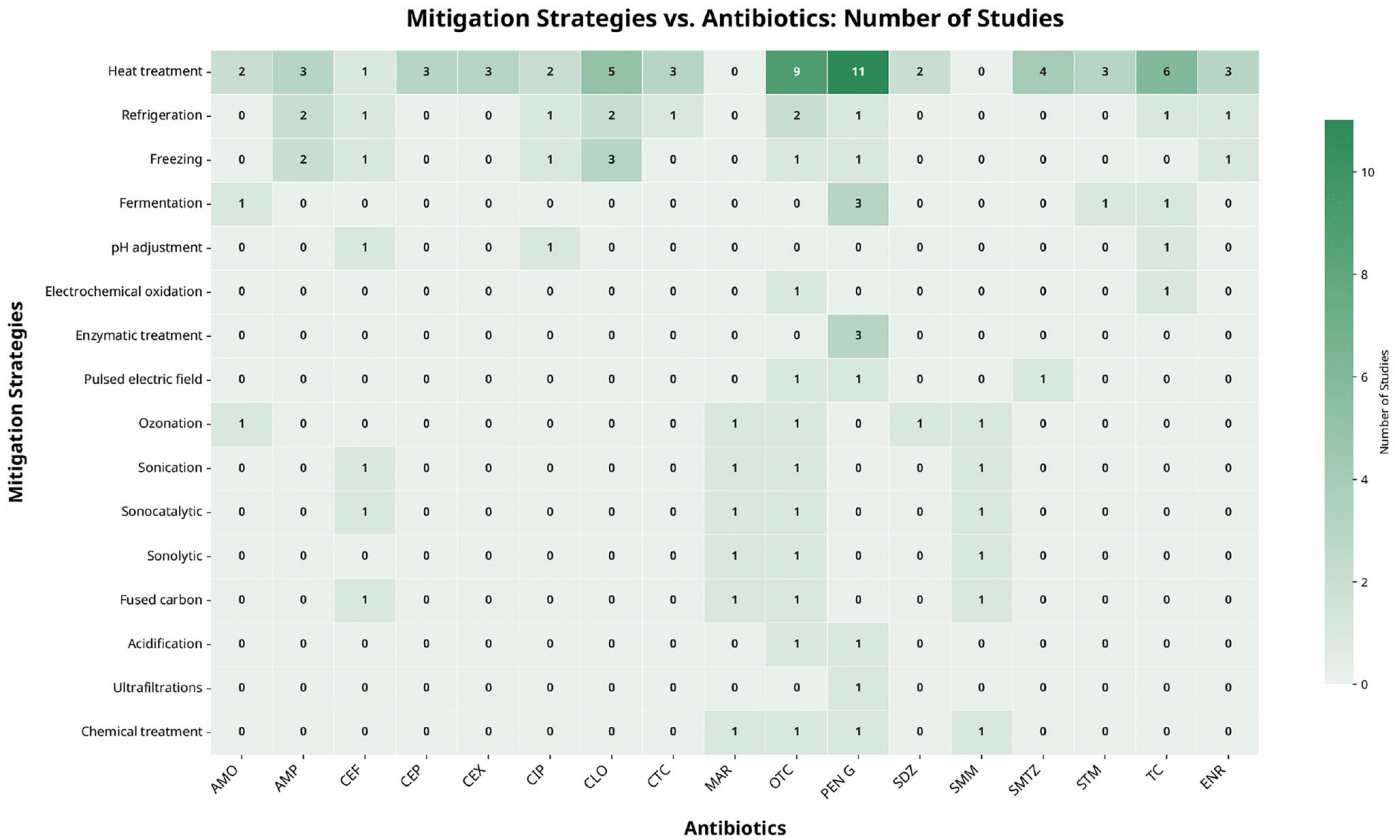
The heat map presents a matrix linking different mitigation strategies (*Y*‐axis) to various types of antibiotics (*X*‐axis). The color intensity (in shades of blue) represents the number of studies applying each approach to a given antibiotic. AMO, amoxicillin; AMP, ampicillin; CEF, ceftiofur; CEP, cefoperazone; CEX, cephalexin; CIP, ciprofloxacin; CLO, cloxacillin; CTC, chlortetracycline; ENR, enrofloxacin; MAR, marbofloxacin; OTC, oxytetracycline; PEN G, penicillin G; SDZ, sulfadiazine; SMM, sulfamonomethoxine sodium; SMTZ, sulfamethazine; STM, streptomycin; TC, tetracycline.

Regarding mitigation strategies, thermal treatment stands out as the most extensively studied and applied method for mitigating ARs in milk, as evidenced by the heat map (Figure 14). This predominance is not coincidental but reflects the proven efficacy and versatility of this approach, as well as its well‐established integration in dairy industrial processes. Particularly notable is the efficiency of heat treatment in mitigating β‐lactam antibiotics such as penicillin G and tetracyclines (OTC, tetracycline, and chlortetracycline), where the highest values (4–16) were observed, suggesting methodological robustness and consistent results. In contrast, low‐temperature preservation strategies, such as refrigeration and freezing, exhibit moderate applicability (values between 1 and 2) for various antibiotics. These results indicate that although these techniques are implemented as complementary approaches in the dairy processing chain, their efficacy in degrading ARs is significantly lower than that of thermal treatment. This difference may be attributed to the fact that low temperatures tend to preserve molecular structures, whereas heat promotes chemical transformations.

Fermentation emerges as a strategy with remarkable selectivity, demonstrating particular efficacy for penicillin G. This pattern suggests a specific interaction between the biochemical mechanisms of lactic fermentation and the molecular structure of β‐lactam antibiotics, possibly related to the ability of certain starter cultures to produce β‐lactamases or other compounds that selectively degrade these antibiotics.

In the context of emerging nonthermal technologies, methods such as ozonation, sonication, sonolysis, and sonocatalysis have demonstrated moderate effectiveness, primarily in targeting antibiotics like MAR, OTC, and sulfamerazine. These results reflect the growing interest in alternative technologies that may offer mitigation efficacy with less impact on the nutritional and organoleptic properties of milk, representing a promising frontier for future research.

Among the less‐reported strategies, PEF, acidification, and ultrafiltration exhibit minimal application, with studies documented for only one or two antibiotics. Similarly, enzymatic treatment and electrochemical oxidation demonstrate high specificity, being applied almost exclusively to penicillin G and tetracyclines (OTC and tetracycline), respectively. This specificity suggests highly selective mechanisms of action, which may be advantageous for targeted applications but limit their utility as universal mitigation strategies.

The overall analysis of the heat map reveals not only the current state of research on the mitigation of ARs in milk but also identifies significant gaps and opportunities for future investigations, particularly in the development and optimization of emerging technologies that could complement or eventually replace conventional thermal methods.

### 3.7 Industrial Feasibility

The transition from promising laboratory‐scale results to viable industrial implementation represents the most significant hurdle for the majority of antibiotic mitigation technologies. The assessment of industrial applicability depends not only on degradation efficiency but also on a multifactorial analysis that encompasses economic and infrastructural barriers, as well as the impact on final product quality. Thermal processing, despite its disadvantages, such as variable efficiency and potential negative impacts on thermosensitive milk components, remains the dominant strategy in the dairy industry. Its predominance is primarily economic: the infrastructure for pasteurization and UHT is already universally integrated into processing plants.

In contrast, the implementation of emerging technologies such as PEF, O_3_, or sonication requires significant capital investment in new equipment, factory redesign, and operator training. The energy costs associated with these technologies can also be substantial. These factors combined make the adoption of emerging technologies an economic challenge, even if they demonstrate high efficiency in the laboratory.

The impact of technology on product quality has direct economic consequences. By degrading an antibiotic, technology may also adsorb or degrade valuable nutrients such as vitamins or essential fatty acids. This can lead to a final product with lower nutritional value, which may be undesirable to consumers.

**FIGURE 15:**
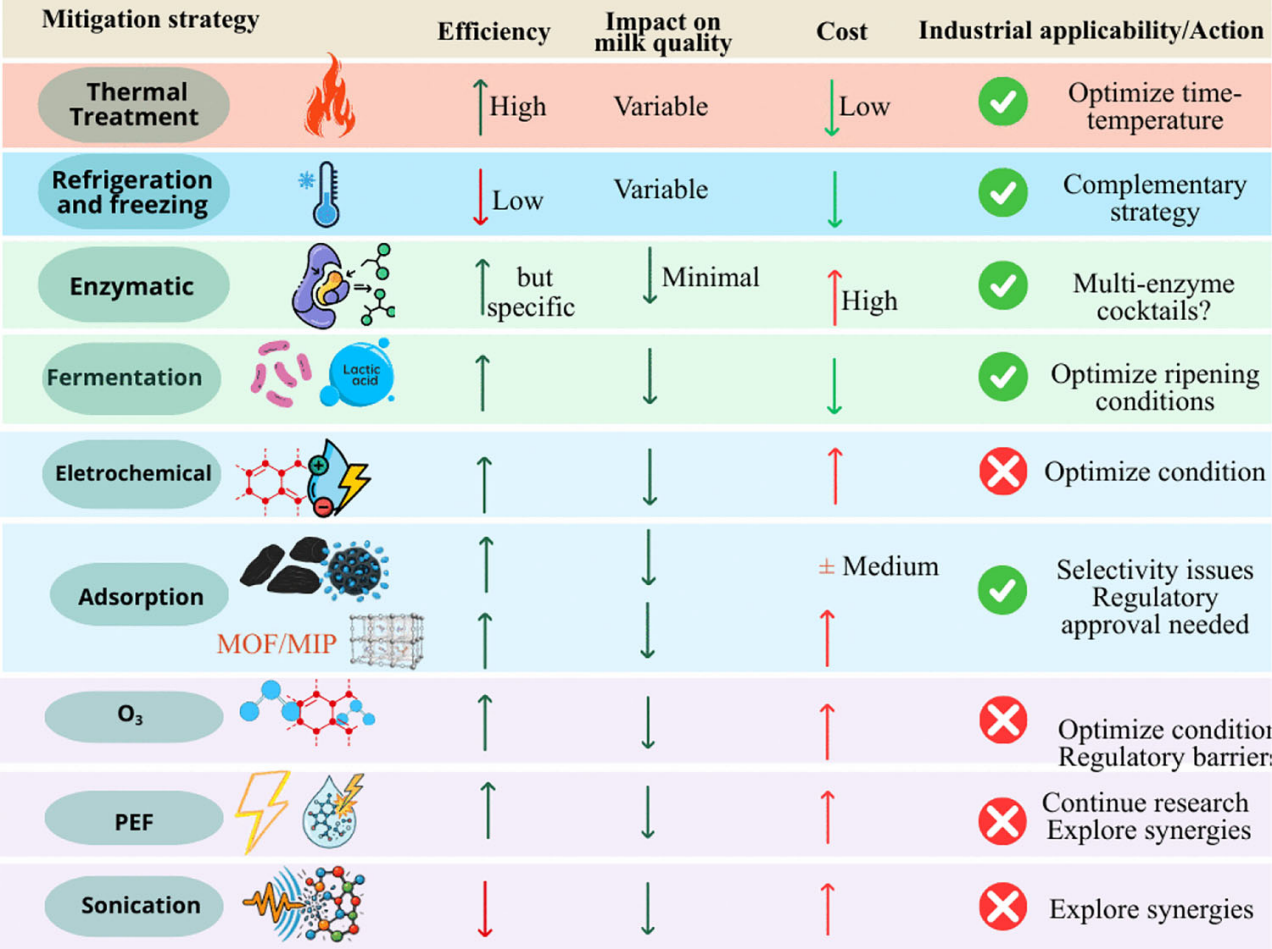
Comparative summary of mitigation strategies for antibiotic residues in milk. Arrows indicate relative levels: ↑ = high, ↓ = low, ± = medium/variable; ✓ = viable, ✗ = not currently.

To summarize, Figure 15 shows a comprehensive trend of mitigation approaches across five key dimensions: efficiency in antibiotic degradation, impact on milk quality, implementation cost, industrial applicability, and recommended actions.

### 3.8 Regulatory Landscape and Food Safety Implications

To ensure adequate consumer protection and uphold the quality of dairy products, the presence of ARs in milk mandates systematic control. Global and regional regulatory bodies have established clear maximum residue limits (MRLs) that must not be exceeded. Key frameworks include the European Union's Commission Regulation No. 37/2010 (EU 2010) and the *Codex Alimentarius* standard CAC/MRL 2–2015 (Codex Alimentarius 2021). Adherence to these MRLs is a legal and public health imperative. However, it must be emphasized that the foundational principle of residue management is prevention, not post‐contamination treatment. The primary focus should be on preventing the occurrence of these residues in milk from the outset. This goal is best achieved through the stringent application of GVP, which encompass the conscious and responsible use of antibiotics and strict compliance with the prescribed withdrawal period for each drug. Consequently, mitigation methods should not be viewed as a primary or elective choice but rather as a corrective measure to be employed only if milk is found to contain ARs. Their role is to salvage contaminated batches, not to serve as a routine step in dairy processing. Despite the satisfactory results presented for some emerging technologies, their path to industrial adoption is fraught with regulatory complexity. As noted, the regulations governing the application of these technologies are very complex and present significant barriers. Several important regulatory issues must be taken into consideration: a primary concern is the potential for mitigation processes to generate by‐products. The toxicity of these newly formed compounds must be thoroughly assessed to ensure they do not pose a greater risk than the original ARs. Currently, few of the emerging technologies discussed have achieved broad regulatory approval for direct application to milk for the specific purpose of degrading ARs.

## 4 Limitations and Robustness of Findings

The included studies lend robustness to this review, as a significant portion of these studies have high JBI scores (> 80%), characterized by methodological rigor that includes adequate controls, validated analytical methods, and appropriate statistical analysis. These works form our baseline of confidence. This is the case for the high efficacy reported for adsorption with modified activated carbon and for ozonation, whose findings are consistently supported by high‐quality studies. In contrast, the majority of the literature, classified as moderate quality (40%–80% JBI), is the main source of uncertainty. For example, the synergistic efficacy of PEF combined with thermal treatment is frequently reported, but many of these studies fail on an essential JBI criterion: the absence of a PEF‐only control group. Without isolating the variable, it is impossible to confidently attribute the degradation to PEF rather than to the thermal component. Similarly, the wide variation in the efficacy of thermal treatment for tetracyclines can be explained by the quality of the analytical method employed; lower‐quality studies using non‐specific bioassays tend to report higher degradation than high‐quality studies using chromatographic methods (LC–MS/MS) capable of distinguishing the parent compound from its metabolites. Finally, low‐quality studies (< 40% JBI), such as some on sonication that lack statistical analysis, are considered unreliable and serve more to illustrate how methodological flaws can lead to misleading conclusions. The low efficacy of sonication as a primary method, however, is a robust conclusion as it is consistent across studies of all quality ranges.

In summary, integrating the JBI assessment allows us to state with greater confidence that the evidence for adsorption and ozonation is strong. The evidence for PEF is promising but should be viewed with caution due to recurrent methodological flaws in the studies. This evidence quality‐centered approach provides a more solid and realistic foundation for recommendations on the future of ARs mitigation technologies in milk.

## 5 Conclusions and Future Perspectives

Although several studies have been published on the antibiotic mitigation strategies in dairy, to our knowledge, this is the first‐ever systematic review based on scientometric analysis. This review reveals that research into the mitigation of ARs in milk is a dynamic and growing field, yet it remains concentrated both geographically and thematically. While thermal treatment continues to be the most studied strategy, its limitations have catalyzed a clear and necessary shift toward emerging nonthermal, biological, and advanced adsorption methods. Our analysis highlights a critical disconnect between laboratory‐scale efficacy and industrial‐scale feasibility, a gap that is further widened by significant regulatory and economic hurdles. The key take‐home messages from this review are:

- Research output is driven more by national research priorities than by milk production volume. Spain leads in publications, while top producers like the USA and India lag, suggesting that targeted funding and regulatory pressure are key drivers.
- Thermal methods offer variable and often insufficient efficacy. While high‐temperature treatments can degrade some residues, they damage milk quality. Common boiling is largely ineffective for many thermostable antibiotics.
- Nonthermal technologies show promise but lack industrial viability. Methods like ozonation and PEF can achieve high degradation rates, but their application is hindered by high costs, challenges in scalability, and the unaddressed safety of degradation by‐products.
- Research is narrowly focused on a few antibiotic classes. The vast majority of studies investigate tetracyclines and β‐lactams, leaving significant knowledge gaps for other clinically important veterinary drugs.

Based on this critical analysis, the future of this field must be guided by a multi‐faceted approach that moves beyond simple degradation efficacy. We propose the following actionable perspectives for research, industry, and policy:

For research:

- Focus on hybrid approaches that combine the advantages of different technologies for the removal of ARs in milk.
- By‐products identification and toxicology, a critical step for regulatory acceptance.
- Research must expand to include a wider range of antibiotic classes beyond tetracyclines and penicillins to reflect the diversity of drugs used in modern veterinary medicine.
- Continued investment in the engineering of food‐grade, highly selective adsorbents and more stable, cost‐effective enzyme systems is crucial for developing practical solutions.

For industry:

- The industry, in collaboration with academia, should invest in pilot‐scale studies to realistically assess the economic and operational feasibility of the most promising emerging technologies.
- Dairy industry stakeholders should initiate early‐stage dialogues with regulatory bodies to clarify the approval pathways for new mitigation technologies and their treated products.
- The industry must continue to invest in and promote Good Agricultural and Veterinary Practices to minimize the initial contamination of milk.

For policymakers:

- Regulatory agencies need to establish clear guidelines for the evaluation and approval of mitigation technologies and the safety assessment of treated foods.
- Funding bodies should prioritize research that addresses the critical gaps identified here, particularly studies on industrial feasibility, by‐product safety, and synergistic approaches.

## Author Contributions

**Emelda Orlando Simbine – Ribisse**: conceptualization, methodology, data curation, writing – original draft, writing – review and editing. **Wilma Custódio Fumo**: data curation, visualization, methodology. **Eugénio da Piedade Edmundo Sitoe**: visualization, data curation. **Patrícia Aparecida de Campos Braga**: writing – review and editing. **Cristiano João Macuamule**: writing – review and editing. **Adriana Pavesi Arisseto Bragotto**: conceptualization, writing – review and editing, supervision.

## Funding

The Organization for Women in Science for the Developing World (OWSD) for the fellowship awarded to E.O.S.R. to undertake Doctoral studies in Brazil.

## Conflicts of Interest

The authors declare no conflicts of interest.
